# FBW7 suppresses ovarian cancer development by targeting the N^6^-methyladenosine binding protein YTHDF2

**DOI:** 10.1186/s12943-021-01340-8

**Published:** 2021-03-03

**Authors:** Fei Xu, Jiajia Li, Mengdong Ni, Jingyi Cheng, Haiyun Zhao, Shanshan Wang, Xiang Zhou, Xiaohua Wu

**Affiliations:** 1 Department of Gynecologic Oncology, Fudan University Shanghai Cancer Center, Fudan University, Shanghai, 200032 China; 2 Department of Oncology, Shanghai Medical College, Fudan University, Shanghai, 200032 China; 3 Fudan University Shanghai Cancer Center and Institutes of Biomedical Sciences, Fudan University, Shanghai, 200032 China

**Keywords:** FBW7, YTHDF2, BMF, N6-methyladenosine, Ubiquitination

## Abstract

**Background:**

The tumor suppressor FBW7 is the substrate recognition component of the SCF E3-ubiquitin ligase complex that mediates proteolytic degradation of various oncogenic proteins. However, the role of FBW7 in ovarian cancer progression remains inadequately understood.

**Methods:**

IP-MASS, co-IP, immunohistochemistry, and western blotting were used to identify the potential substrate of FBW7 in ovarian cancer. The biological effects of FBW7 were investigated using in vitro and in vivo models. LC/MS was used to detect the m^6^A levels in ovarian cancer tissues. MeRIP-Seq and RNA-Seq were used to assess the downstream targets of YTHDF2.

**Results:**

We unveil that FBW7 is markedly down-regulated in ovarian cancer tissues and its high expression is associated with favorable prognosis and elevated m^6^A modification levels. Consistently, ectopic FBW7 inhibits ovarian cancer cell survival and proliferation in vitro and in vivo, while ablation of FBW7 empowers propagation of ovarian cancer cells. In addition, the m^6^A reader protein, YTHDF2, is identified as a novel substrate for FBW7. FBW7 counteracts the tumor-promoting effect of YTHDF2 by inducing proteasomal degradation of the latter in ovarian cancer. Furthermore, YTHDF2 globally regulates the turnover of m^6^A-modified mRNAs, including the pro-apoptotic gene BMF.

**Conclusions:**

Our study has demonstrated that FBW7 suppresses tumor growth and progression via antagonizing YTHDF2-mediated BMF mRNA decay in ovarian cancer.

**Supplementary Information:**

The online version contains supplementary material available at 10.1186/s12943-021-01340-8.

## Background

Epithelial ovarian cancer (EOC) is one of the most common causes of cancer-related mortality in women worldwide [1]. Despite the advances in treatments with surgery and targeted chemotherapy [2], the prognosis has only marginally improved because of the relapse and chemotherapy resistance [3]. Therefore, thorough understanding of the pathogenic mechanisms of EOC is of great importance to develop new treatment strategies and ameliorate the overall prognosis of the EOC patients.

F-box and WD repeat domain-containing 7 (FBW7), also known as FBXW7 or hCDC4, is encoded by the *FBXW7* gene residing at chromosome 4q31 which is deleted in ~ 30% of human cancers [4]. It belongs to the F-box protein family and acts as the substrate recognition component of the Skp1-Cullin-F-box (SCF) ubiquitin ligase complex. FBW7 selectively mediates ubiquitination and proteasomal degradation of oncogenic proteins, such as c-Myc, Cyclin E, c-Jun, Notch1, mTOR [5], and is therefore regarded as a tumor suppressor. FBW7 was also shown to be transcriptionally induced by and synergize with p53 in maintaining genomic stability and suppressing carcinogenesis [6–8]. Several genetic models have elegantly demonstrated FBW7’s tumor inhibitory activity in vivo [9]. Although loss of two alleles of *Fbxw7* causes embryonic lethality of mice because of severe abnormalities in vascular development [10], heterozygous inactivation or conditional knockout of this gene leads to profound chromosomal instability, upregulation of its targeting oncoproteins and increased tumor burden [7, 8, 11–14]. Also, FBW7 is able to facilitate nonhomologous end-joining (NHEJ) repair through the lysine 63-linked polyubiquitylation, but without degradation, of XRCC4 [15]. Consistent with its role as a tumor suppressor, the mutation of the *FBXW7* gene was found in around 6% of all human cancers [4], which not only abrogates the tumor suppressive activity of FBW7, but also endows these mutants with the oncogenic function [13, 16]. Additionally, FBW7 was found to be ubiquitously downregulated in human cancers which is related to unfavorable prognosis [17]. The downregulation of FBW7 in tumors might be associated with the frequent mutation of the *TP53* gene, and low FBW7 expression level along with *TP53* mutation predicts distinctively poor prognosis in gastric cancer [18]. In ovarian cancer, it was found that the frequency of the *FBXW7* gene mutation is approximately 2.5 ~ 8.3% [19, 20] and the downregulation of FBW7 expression can be attributed to the *TP53* gene mutation, particularly, in the serous carcinoma samples with the *TP53* mutation frequency of ~ 95% [21–23]. However, the role of FBW7 in the progression of ovarian cancer remains incompletely understood.

To unravel this question, we performed a set of bioinformatics and experimental analyses to dissect the role of FBW7 in ovarian cancer. In addition, a co-immunoprecipitation assay combined with mass spectrometry analysis was conducted and the human YTH domain family 2 (YTHDF2), a N^6^-methyladenosine (m^6^A) binding protein, was identified as an interacting protein as well as a substrate of FBW7. YTHDF2 is an m^6^A reader that selectively binds to the m^6^A consensus motif and promotes mRNA decay [24, 25]. The YTHDF2 encoding gene was found to be overexpressed and responsible for development of acute myeloid leukemia (AML) [26, 27], whereas it turned into a tumor suppressor by targeting EGFR, IL11 and SERPINE2 mRNAs for degradation in hepatocellular carcinoma [28, 29]. Very recently, YTHDF2 was found to be degraded by the E3 ubiquitin ligase complex consisting of Cullin 1 (CUL1), Cullin 4A (CUL4A), damaged DNA-binding protein 1 (DDB1), and S-phase kinase-associated protein 2 (SKP2) [30]. We reveal here that overexpression of YTHDF2 prompts ovarian cancer cell propagation by globally modulating mRNA turnover through m^6^A modification, which is associated with worse overall survival in patients. Furthermore, FBW7 interacts with and promotes proteolytic degradation of YTHDF2, thereby stabilizing the pro-apoptotic BMF mRNA and evoking apoptosis of ovarian cancer cells. Collectively, our study as detailed below demonstrates the role of the FBW7-YTHDF2-BMF cascade in suppression of ovarian cancer.

## Methods

### The human specimens and cell lines

Totally 120 Chinese patients diagnosed with high-grade serous ovarian carcinoma and 10 Chinese patients with ovarian cyst were involved in this study. All patients had surgical resection at Fudan University Shanghai Cancer Center (FUSCC). The tissue samples were collected immediately after surgery and stored in preservation buffer at − 80 °C. Informed consent was obtained from all patients, and the use of clinical samples in this study was approved by the ethics committee of FUSCC. Ovarian cancer cell lines used in this study, SKOV3, OVCA420, OVCA429 and OVCAR8 were obtained from ATCC.

### Establishment of stable cell lines

The specific shRNAs targeting FBXW7 or YTHDF2 (Supplementary Table S1) were cloned into the lentiviral vector pLKO.1 (Sigma-Aldrich, St louis, MO, USA). The specific plasmid overexpressing FBW7 or YTHDF2 was generated by inserting the full-length cDNA amplified by PCR into the lentiviral vector pCDH (Sigma-Aldrich). The 293 T cells were transfected with pCDH or pLKO.1 carrying the specific sequences, along with the packaging plasmids, psPAX2 and pMD2.G. The virus particles were generated and collected 48 h post-transfection and used to infect ovarian cancer cell lines which were then selected by puromycin (Sigma-Aldrich) for 3 days. Knockdown or overexpression efficiency was confirmed at both mRNA and protein levels.

### Quantitative reverse transcription and quantitative RT-PCR (RT-qPCR)

Total RNA was extracted from ovarian cancer samples or cell lines by using TRIzol reagent (Life Technologies, Waltham, MA, USA). Complementary DNA (cDNA) synthesis was performed with PrimeScript RT reagent Kit (Takara, Japan) using ~ 1 μg RNA each sample. RT-qPCR reactions were conducted using TB Green Premix (Takara, Japan). The primers for FBXW7, YTHDF2, BMF, KCNK3, MYBL2, LEMD1, SIRPD, LY6K, SEMA3C, C1QTNF1, ZDHHC14 and MOCS3 are listed in Supplementary Table S1.

### Immunoblotting (IB)

Cells were harvested and lysed with RIPA buffer. Proteins were extracted and loaded in SDS-PAGE, and transferred onto PVDF membrane (Millipore, Billerica, MA, USA). After blocking with Bovine Serum Albumin (Beyotime, China) and sequential incubation with the primary and secondary antibodies, the blots were detected using the ECL detection kit (Millipore). The anti-FBW7 antibody was purchased from Bethyl Laboratories (Montgomery, TX, USA), and the anti-YTHDF2 and anti-GAPDH antibodies were purchased from Proteintech (Wuhan, Hubei, China).

### Co-Immunoprecipitation (co-IP)

Cells were harvested and lysed with mild RIPA buffer directly on plate for 30 min. Meanwhile, 50 μl dynabeads protein G (Life Technologies) were incubated with 3 μg antibody at room temperature for 1 h. Then mix the protein lysate with the beads-antibody complex and incubate overnight at 4 °C. Beads were washed three times with lysis buffer. Bound proteins and 10% inputs were detected by IB.

### Immunofluorescence staining (IF)

2 × 104 HeyA8 or OVCAR8 cells were seeded on coverslips in a 24-well dish and further incubated for 12 h. Cells were fixed with 4% paraformaldehyde for 15 min, then incubated with 0.5% Triton X-100 in PBS for 15 min, and washed three times in PBS. After blocking in 1% BSA for 30 min, the cells were incubated with rabbit anti-YTHDF2 antibody Proteintech (Wuhan, Hubei, China) and mouse anti-FBW7 antibody (Santa Cruz Biotechnology, CA, USA) overnight at 4 °C. After washing several times in PBS, the coverslips were incubated with anti-mouse secondary antibody conjugated with Alexa 488 and Cy3-conjugated anti-rabbit secondary antibody for 30 min. After washing several times in PBS, the Slides were mounted and sealed with DAPI Staining Solution. The images were acquired using a confocal microscope (Leica, Wetzlar, Germany).

### Immunohistochemistry (IHC)

Immunohistochemistry was performed using a primary antibody against YTHDF2 antibody Proteintech (Wuhan, Hubei, China) and FBW7 antibody (Bethyl Laboratories, Montgomery, TX, USA). The immunostaining results were examined by two researchers independently. Immunohistochemical evaluation was based on the intensity and percentage of membranous and cytoplasmic reactivity. A 10% expression threshold was defined as positive. Then, no positive cells, less than 10% of positive cells, 10–50% of positive cells, more than 51% of positive cells were defined as negative, weak, moderate, strong expression respectively. Tumors with moderate and strong staining were regarded as high expression.

### In vivo ubiquitination assay

SKOV3 cells were transfected with different combinations of plasmids encoding FBW7, YTHDF2 and His-Ub. After 48 h, cells were harvested and divided into two parts, one for IB and the other for the ubiquitination assay. Briefly, cell pellets were lysed with buffer I (8 M urea, 0.1 M Na2HPO4/ NaH2PO4 (pH 8.0), 10 mM Tris-HCl (pH 8.0), 10 mM β-mercaptoethanol, 5 mM Imidazole) and incubated with Ni-NTA beads (Takara, Japan) for 6 h at room temperature. Beads were washed twice with buffer I, and twice with buffer II (8 M urea, 0.1 M Na2HPO4/NaH2PO4 (pH 6.3), 10 mM Tris-HCl (pH 6.3), 10 mM β-mercaptoethanol). The bound protein complex was eluted and analyzed by IB.

### In vitro cell proliferation, colony formation, and anchorage-independent cell growth assays

For the cell proliferation assay, 2000 cells were seeded into 96-well plates, cell viability was assessed for 5 consecutive days by the Cell Counting Kit-8 (CCK-8) (Dojindo, Japan). For the colony formation assay, 1000 cells were seeded into 6-well plates for 2 weeks, and colonies were stained with crystal violet and counted. For the anchorage-independent cell growth assay, 20,000 cells were seeded into 6-well plates coated with soft agar for 4 weeks, and the growing colonies were counted under the microscope. The colony formation assay was used to evaluate the long-term cell growth on a solid surface, while the anchorage-independent cell growth assay was employed to assess the ability of transformed cells to grow independently of a solid surface.

### In vivo mouse xenograft study

In vivo experiments were all conducted using 5-week old Female BALB/c nude mice (Shanghai SLAC Laboratory Animal Co., Ltd). 5 × 10^5^ cells were suspended in 100 μl PBS and then were inoculated subcutaneously. After the tumors were formed, the tumor volumes were measured every 3 days. Mice were sacrificed when the tumor diameter reached about 15 cm, and the tumors were excised and weighted. Tumor volume was calculated based on the formula: volume = length × width^2^ × 0.5. Animal experiments were all performed according to the Control of Department of Laboratory Animal Science in Shanghai Medical College of Fudan University and the animal ethic principles.

### RNA m^6^A quantification by LC-MS/MS

RNA m^6^A quantification by LC-MS/MS was conducted as described previously [31]. Briefly, total RNAs were isolated using TRIzol reagent (Life Technologies). 200 ng mRNA was incubated with nuclease P1 (Sigma-Aldrich) in 20 μl buffer containing 25 mM NaCl, 2.5 mM ZnCl_2_ at 37 °C for 2 h, then added 2.2 μl NH_4_HCO_3_ (1 M) and alkaline phophatase (Sigma-Aldrich), and incubated at 37 °C for 2 h. Following centrifugation at 13000 rpm for 10 min at 4 °C, 10 μl of the solution was analyzed by LC-MS/MS at Mass Spectrometry Application Research Center of the Institutes of Biomedical Sciences in Fudan University. The expression level of m^6^A was dichotomized for OS before the log-rank test according to optimal cutoff values calculated by the “surv_cutpoint” function of the “survminer” R package.

### Analysis of BMF mRNA stability

Cells were incubated with actinomycin D (5 μg/ml) for 0 h, 3 h or 6 hoursfollowed by RNA extraction. The half-life of BMF mRNA was analyzed by quantitative RT-PCR. For the 3-deazaadenosine (DAA) treatment assay, cells were incubated with 50uM DAA followed by RNA extraction. The level of BMF mRNA was analyzed by quantitative RT-PCR.

### Library preparing and RNA sequencing

Transcriptome sequencing was performed by Cloud-Seq Biotech (Shanghai, China). RNA libraries were constructed by adopting the NEBNext® Ultra™ II Directional RNA Library Prep Kit (New England Biolabs, Inc., MA, USA). Library sequencing was performed on the illumina Hiseq instrument with 150 bp paired end reads. After harvesting the Paired-end reads and quality controlled by Q30 and cutadapt software (v1.9.3), the high quality clean reads were aligned to the reference genome (UCSC hg19) with the hisat2 software (v2.0.4). Then, the gene level FPKM as the expression profiles of mRNA was analyzed by the Ensembl gtf gene annotation file and the cuffdiff software (part of cufflinks), the fold change and *p*-value were calculated according to FPKM, and the differentially expressed mRNAs with statistical significance were obtained. GO and Pathway enrichment analysis were performed according to the differentially expressed mRNAs.

### m^6^A-RNA immunoprecipitation (MeRIP) assay and m^6^A sequencing

Polyadenylated RNAs were prepared and sonicated into fragments of 100–200 nt. A small portion of the RNA fragments was saved as input samples. MeRIP was performed as previously described [32]. Briefly, 4 μg fragmented RNAs were incubated with 2 μg anti-m^6^A antibody (Synaptic Systems, Goettingen, Germany) in 1 x IP buffer (10 mM TrisHCl, pH 7.4, 150 mM NaCl, 0.1% NP-40) for 2 h at 4 °C. The m^6^A-IP complex was then incubated with Dynabeads protein A (Life Technologies) for 2 h at 4 °C. The bound RNAs were washed and eluted through competition with N6-methyladenosine (Santa Cruz Biotechnology, CA, USA) and then purified by the RNA cleanup kit (Zymo Research, CA, USA). The NEBNext® Ultra II Directional RNA Library Prep Kit (New England Biolabs, MA, USA) was used for library construction. Library sequencing was performed on an illumina Hiseq instrument with 150 bp paired-end reads by Cloudseq Biotech Inc. (Shanghai, China).

### Statistical analysis

Statistical analysis was conducted by the SPSS software (IBM) and GraphPad Prism software (version 6). The differences between two groups or more than two groups were compared using Student’s t-test. Survival analyses were conducted by Kaplan-Meier curve and log-rank test. The linear regression test was used to analyze the genes expression correlation. *p* < 0.05 was regarded as statistically significant. All the data are showed as mean ± SD.

## Results

### Downregulation of FBW7 is associated with unfavorable prognosis and decreased m^6^A modification levels in human ovarian cancer

**Fig. 1:**
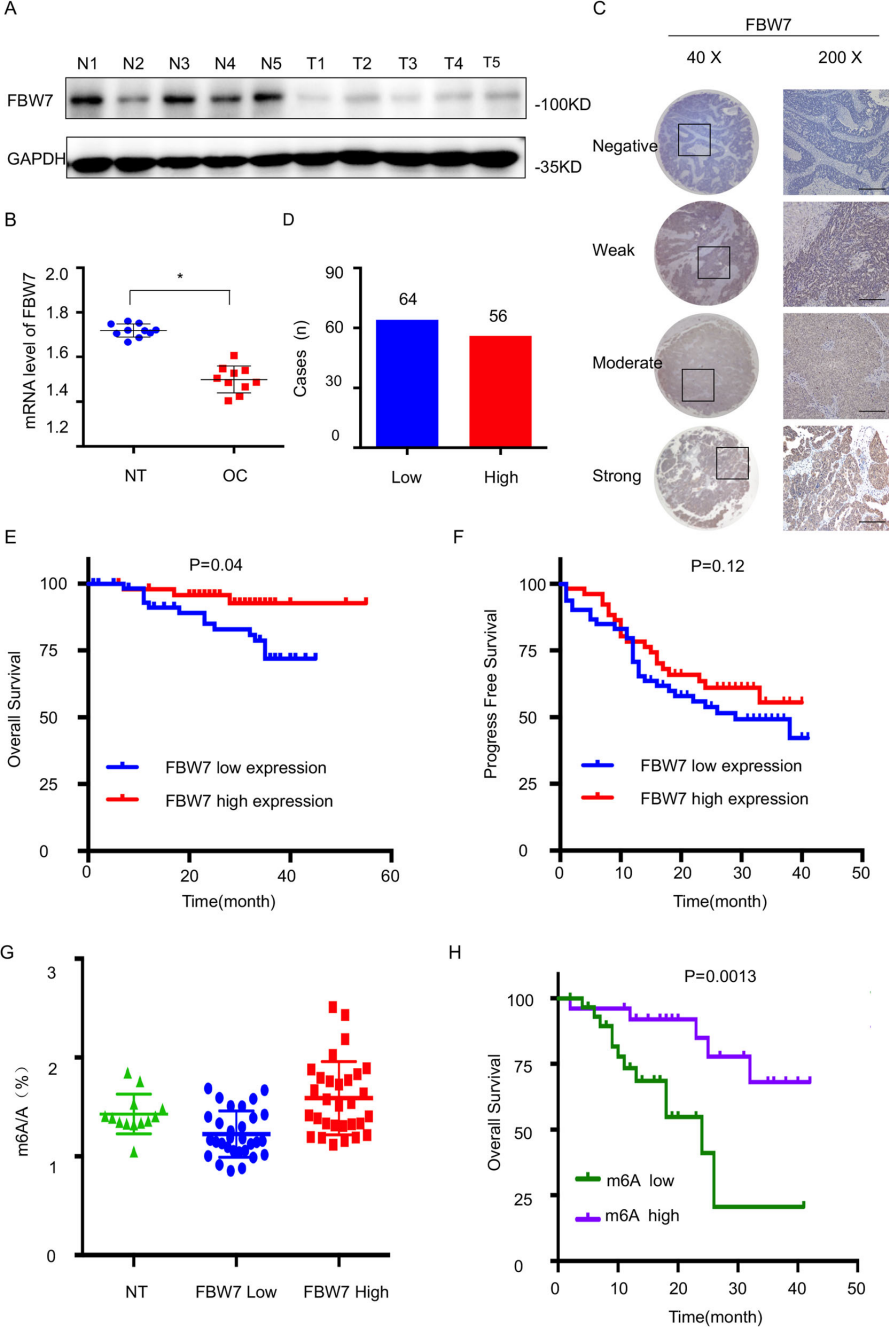
Frequent downregulation of FBW7 in human epithelial ovarian cancer. **a** Expression of FBW7 in cancerous and normal ovarian tissues detected by IB. **b** Expression of FBW7 in cancerous and normal ovarian tissues analyzed by RT-qPCR. **c**-**d** Expression of FBW7 by IHC staining of human ovarian cancer tissue arrays. Scale bar, 200 μm. **e** FBW7 downregulation is significantly associated with shorter overall survival in epithelial ovarian cancer patients. **f** No significant correlation is observed between FBW7 expression and progression-free survival. **g** Quantification of m^6^A levels in 13 normal ovarian specimens and 60 ovarian cancer tissues classified into FBW7 high expression and low expression groups. **h** Higher level of m^6^A levels is significantly associated with better overall survival in epithelial ovarian cancer patients. m^6^A high: m^6^A ≥ 1.04%, m^6^A low: m^6^A < 1.04%. Results were presented as mean ± SD of three independent experiments. *P < 0.05 or **P < 0.01 indicates a significant difference between the indicated groups

Since a paucity of evidence had been found on the role of FBW7 in ovarian cancer, we first determined its clinical implication by comparing the expression of FBW7 in both cancerous and non-cancerous ovarian tissues. FBW7 was markedly downregulated in ovarian cancer tissues (Fig. 1a, b and Supplementary Fig. 1A). Consistently, FBW7 was also found to be significantly reduced in the TCGA RNA-Seq dataset of 586 ovarian cancer samples (Supplementary Figure 1B). Next, the FBW7 expression in the ovarian cancer tissue microarrays, containing 120 tumor samples, was analyzed by immunohistochemistry (IHC) staining and as thus the ovarian cancer specimens could be categorized into two groups with low or high expression level of FBW7 (Fig. 1c and d). We then explored the relationship between FBW7 expression and the clinicopathological features of ovarian cancer. However, no significant correlation was observed between FBW7 expression and age, tumor stage, ascites, lymph node metastasis or menopause (Supplementary Table S2; *P* > 0.05). The Kaplan-Meier survival analysis showed that high level of FBW7 is significantly associated with favorable overall survival in ovarian cancer (Fig. 1e, *P* = 0.04), which is consistent with the result from the TCGA ovarian cancer cohort (Supplementary Fig. 1C). Additionally, high level of FBW7 was moderately, but not significantly, correlated with better progress-free survival (PFS) (Fig. 1f), probably due to the limited sample size. Therefore, these observations indicate that FBW7 is a favorable prognostic marker that may suppress ovarian tumorigenesis and progression.

FBW7 was a typical tumor suppressor, mainly involved in the process of ubiquitylation and degradation of many onco-proteins. N^6^-methyladenosine (m^6^A) has emerged as an abundant reversible modification, which has been reported to play critical roles in physiological and pathological processes. However, the regulation of the m^6^A modification system remains elusive. Here we measured m^6^A levels in 60 human ovarian cancer tissue samples. According to the optimal cutoff point calculated by “survminer” R package, samples with a m^6^A expression no more than 0.01 were considered as the m^6^A low expression population, while the rest was considered as the m^6^A high expression population. Interestingly, m6A levels were elevated in patients with tumors expressing higher levels of FBW7 (Fig. 1g), and better overall survival was significantly correlated with higher levels of m^6^A (Fig. 1h), which suggested FBW7 might be an upstream regulator for the m^6^A modification system.

### FBW7 inhibits ovarian cancer cell propagation as a tumor suppressor

**Fig. 2:**
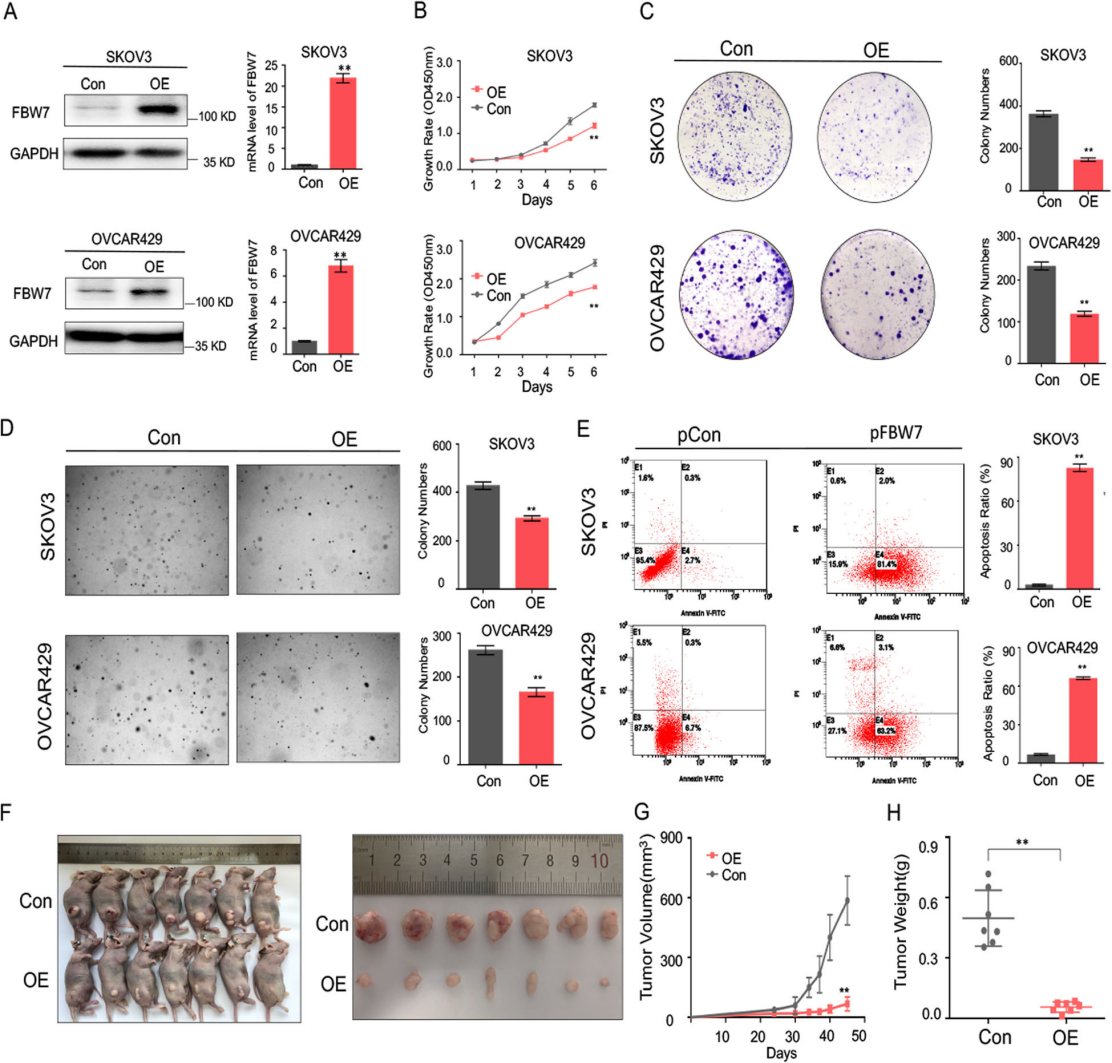
Overexpression of FBW7 significantly suppresses ovarian cancer cell proliferation and growth in vitro and in vivo. **a** Stable overexpression of FBW7 in SKOV3 and OVCAR429 cells. The expression of FBW7 is verified at both mRNA and protein levels. **b** Overexpression of FBW7 significantly reduces the proliferation rate of SKOV3 and OVCAR429 cells. **c** Overexpression of FBW7 impairs the colony-forming ability of ovarian cancer cells. **d** Overexpression of FBW7 suppresses anchorage-independent growth ovarian cancer cells. **e** Overexpression of FBW7 promotes ovarian cancer cell apoptosis. **f** Overexpression of FBW7 in SKOV3 cells markedly suppresses ovarian tumor growth in the xenograft mice. **g** The size of tumors formed in the xenograft mice is monitored every 3 days. **h** Overexpression of FBW7 significantly reduces weight of tumors in the xenograft mice. Results were presented as mean ± SD of three independent experiments. *P < 0.05 or **P < 0.01 indicates a significant difference between the indicated groups

To investigate the biological function of FBW7 in ovarian cancer, we generated FBW7-stably overexpressing SKOV3 and OVCAR429 cell lines, both of which sustain low expression level of endogenous FBW7 (Fig. 2a, Supplementary Fig. 2). Overexpression of FBW7 significantly suppressed ovarian cancer cell proliferation (Fig. 2b), colony-forming ability (Fig. 2c), and anchorage-independent cell growth (Fig. 2d). The tumor inhibitory activity could be attributed to the induction of apoptosis, as ectopic FBW7 strikingly augmented the Annexin V-positive cell population (Fig. 2e). Furthermore, we established a xenograft mouse model to test if FBW7 suppresses ovarian tumor growth in vivo. In accordance with the cell-based results, ectopic FBW7 markedly repressed tumor growth in mice (Fig. 2f), as reflected by the significant inhibition of the tumor volume and weight when compared to the control group (Fig. 2g and h). In addition, we also generated FBW7-depleted HeyA8 and OVCAR8 ovarian cancer cell lines using two independent shRNAs (Supplementary Fig. 3A). Consistently, ablation of FBW7 significantly promoted ovarian cancer cell proliferation (Supplementary Fig. 3B), colony-forming ability (Supplementary Fig. 3C), and anchorage-independent cell growth (Supplementary Fig. 3D). Taken together, these results demonstrate that FBW7 plays a tumor suppressive role in ovarian cancer.

### FBW7 induces ubiquitination and proteasomal degradation of YTHDF2

**Fig. 3:**
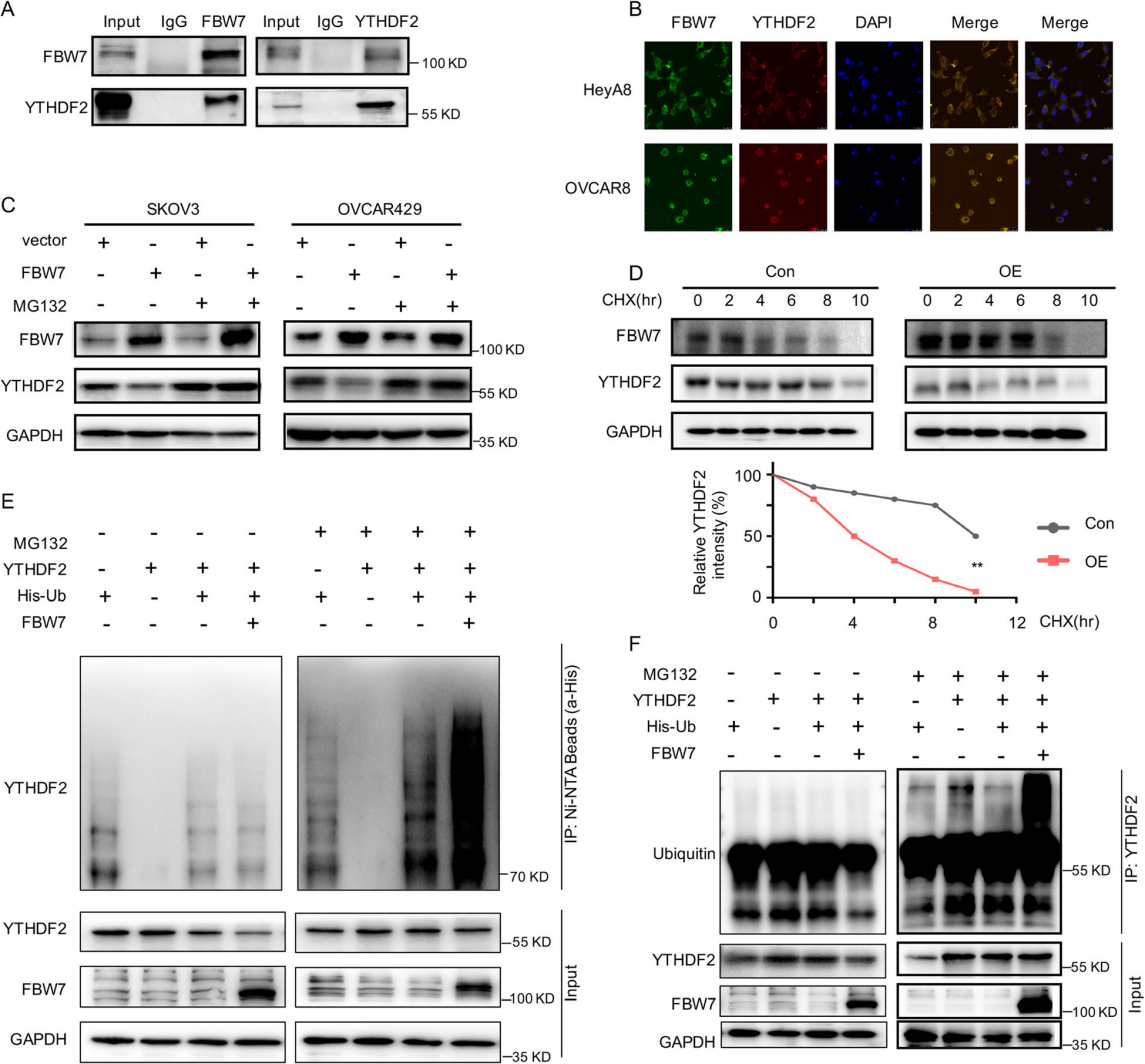
FBW7 binds to and mediates the proteolytic degradation of YTHDF2 in ovarian cancer. **a** FBW7 interacts with YTHDF2 by the reciprocal co-IP assays in SKOV3 cancer cells. **b** The IF staining indicates the co-localization of FBW7 and YTHDF2 in the cytoplasm. **c** Ectopic expression of FBW7 reduces the YTHDF2 protein levels in SKOV3 and OVCAR429 cells, which can be blocked by the proteasome inhibitor MG132 (20 μM). **d** The half-life of YTHDF2 is shortened upon FBW7 overexpression. The cells were treated with 100 mg/ml of cycloheximide (CHX) and harvested at different time points as indicated. The quantification of YTHDF2 expression is shown in the lower panel. Overexpression of FBW7 shortened the half-life of YTHDF2 from 7.0 h to 3.3 h. **e** FBW7 promotes ubiquitination of YTHDF2 in 293 T cells. Cells were transfected with combinations of plasmids encoding YTHDF2, FBW7 or His-Ub as indicated, and treated with MG132 (20 μM) or DMSO for 6 h before harvested for in vivo ubiquitination assay. Bound and input proteins were detected by IB assays with the indicated antibodies. **f** The ubiquitination of YTHDF2 is mediated by FBW7. Cells were transfected with combinations of plasmids encoding YTHDF2, FBW7 or His-Ub as indicated, and treated with MG132 (20 μM) or DMSO for 6 h before harvested for the IP assays using the anti-YTHDF2 antibody. Results were presented as mean ± SD of three independent experiments. *P < 0.05 or **P < 0.01 indicates a significant difference between the indicated groups

To decipher the molecular mechanism behind FBW7-mediated regulation of the m^6^A modification system, we conducted a co-IP assay using the anti-FBW7 antibody coupled with mass spectrometry analysis to identify the FBW7-interacting proteins in the FBW7-stably overexpressing SKOV3 cells (Supplementary Fig. 4A-C, Supplementary table 3). This screening led to identification of YTHDF2 as one of the FBW7-binding proteins. To validate this interaction, we performed the reciprocal co-IP assays and indeed verified that FBW7 binds to YTHDF2 in SKOV3 cancer cells (Fig. 3a). The immunofluorescence (IF) staining suggested that the interaction may occur in the cytoplasm (Fig. 3b). Since FBW7 is a component of the SCF E3-ubiquitin ligase, we sought to determine if FBW7 regulates YTHDF2 protein stability through their physical interaction. By introducing the FBW7-encoding plasmid into SKOV3 and OVCAR429 cells, we found that the protein level of YTHDF2 dramatically declined upon FBW7 overexpression, while this reduction is completely restored by treatment of cells with the proteasome inhibitor MG132 (Fig. 3c). Additionally, overexpression of FBW7 shortened the half-life of YTHDF2 protein as indicated by the cycloheximide-chase experiment (Fig. 3d). In line with these results, we also showed that FBW7 drastically enhances YTHDF2 ubiquitination (Fig. 3e), which was validated by another set of ubiquitination assays using the anti-YTHDF2 antibody (Fig. 3f). Therefore, these findings demonstrate that FBW7 prompts ubiquitination-mediated proteolytic degradation of YTHDF2 in ovarian cancer to regulate the m^6^A modification system.

### YTHDF2 is negatively correlated with FBW7 expression and prognosis of ovarian cancer

**Fig. 4:**
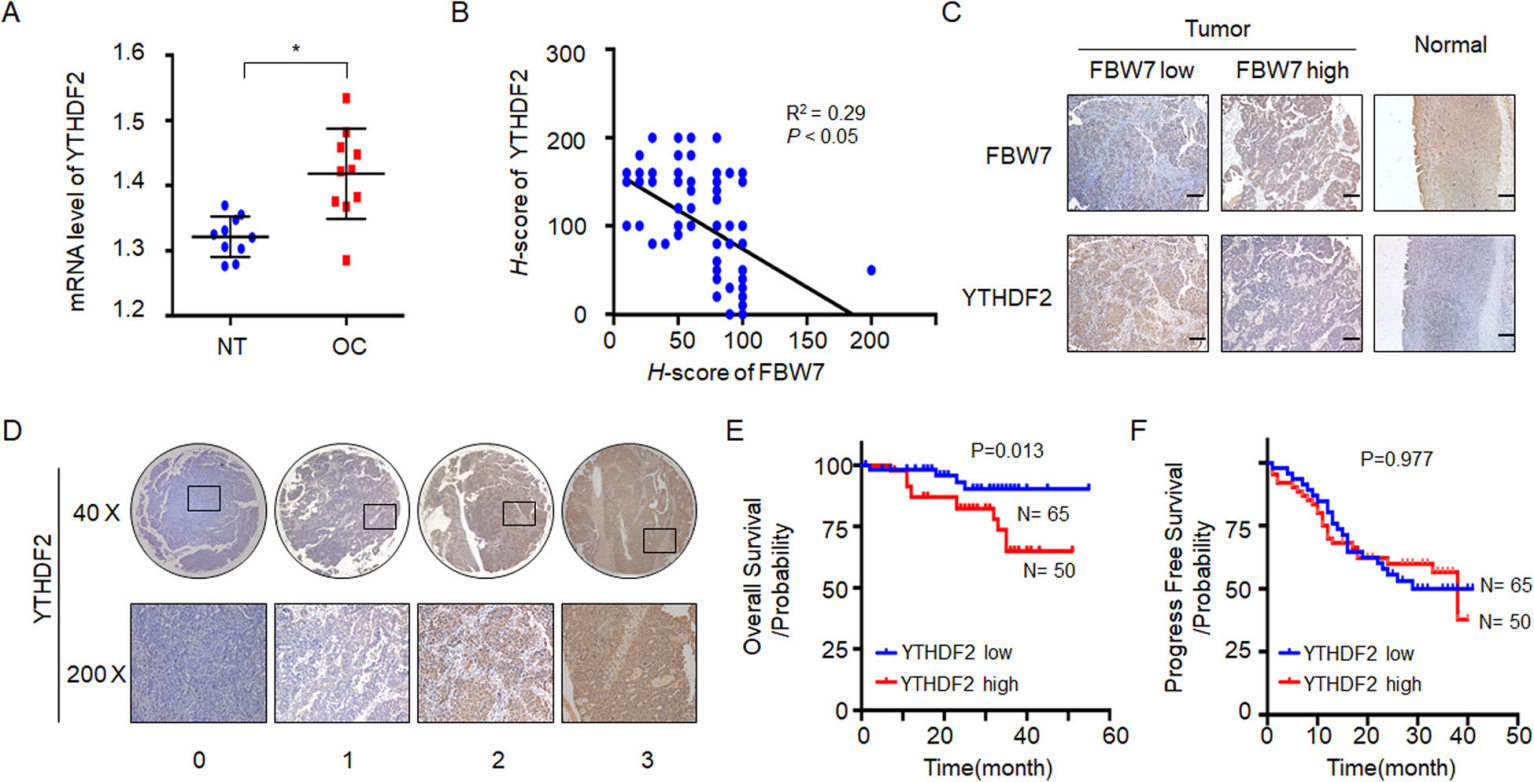
Upregulation of YTHDF2 is negatively associated with FBW7 expression and predicts unfavorable prognosis. **a** Expression of YTHDF2 in cancerous and non-cancerous ovarian tissues detected by RT-qPCR. **b**-**c** IHC staining of human the ovarian cancer tissue arrays shows that YTHDF2 expression is negatively correlated with FBW7 expression. **d** Expression of YTHDF2 by IHC staining of the human ovarian cancer tissue arrays. Scale bar, 200 μm. **e** YTHDF2 upregulation is significantly associated with better overall survival in ovarian cancer patients. **f** No significant correlation is observed between YTHDF2 expression and progression-free survival. * *P* < 0.05

As YTHDF2 is a substrate of the tumor suppressor FBW7 for degradation, we inquired if the inverse correlation between the two proteins also exists in primary ovarian cancer tissues. First, we analyzed the expression of YTHDF2 in the cancerous and non-cancerous ovarian tissues, and found that YTHDF2 is frequently elevated in ovarian cancer (Fig. 4a). Consistently, YTHDF2 was significantly upregulated in ovarian cancer samples versus normal ovarian tissues in the TCGA database (Supplementary Fig. 5A). We then examined YTHDF2 expression using the ovarian cancer tissue microarrays as mentioned above (Fig. 1c) and indeed found a negative correlation between the expression of YTHDF2 and FBW7 (Fig. 4b and c), which is in agreement with the results illustrated in Fig. 3c-f. According to the expression level of YTHDF2, these tumor specimens were categorized into two groups (Fig. 4d) and the clinicopathological features of each group were analyzed. However, no significant correlation was observed between YTHDF2 expression and age, tumor stage, ascites or lymph node metastasis (Supplementary table 4, Supplementary table 5; *P* > 0.05). The Kaplan-Meier survival analysis displayed that increased expression of YTHDF2 is significantly correlated with worse overall survival (OS) in ovarian cancer (Fig. 4e, *P* = 0.0013), which is in line with the result from the TCGA ovarian cancer cohort (Supplementary Fig. 5B). Nevertheless, YTHDF2 is not an appropriate prognostic marker for the progress-free survival (PFS) (Fig. 4f). And the OS of the FBW7 high expression /YTHDF2 low expression group was better than that of the BW7 low expression /YTHDF2 high expression group (Supplementary Fig. 13). In multivariate analysis, YTHDF2 expression was adverse independent prognosticators for OS (Supplementary table 6). Thus, these observations strongly suggest that YTHDF2 may promote survival and growth of ovarian cancer, which is addressed as follows.

### YTHDF2 endorses ovarian cancer development as an oncogenic protein

**Fig. 5:**
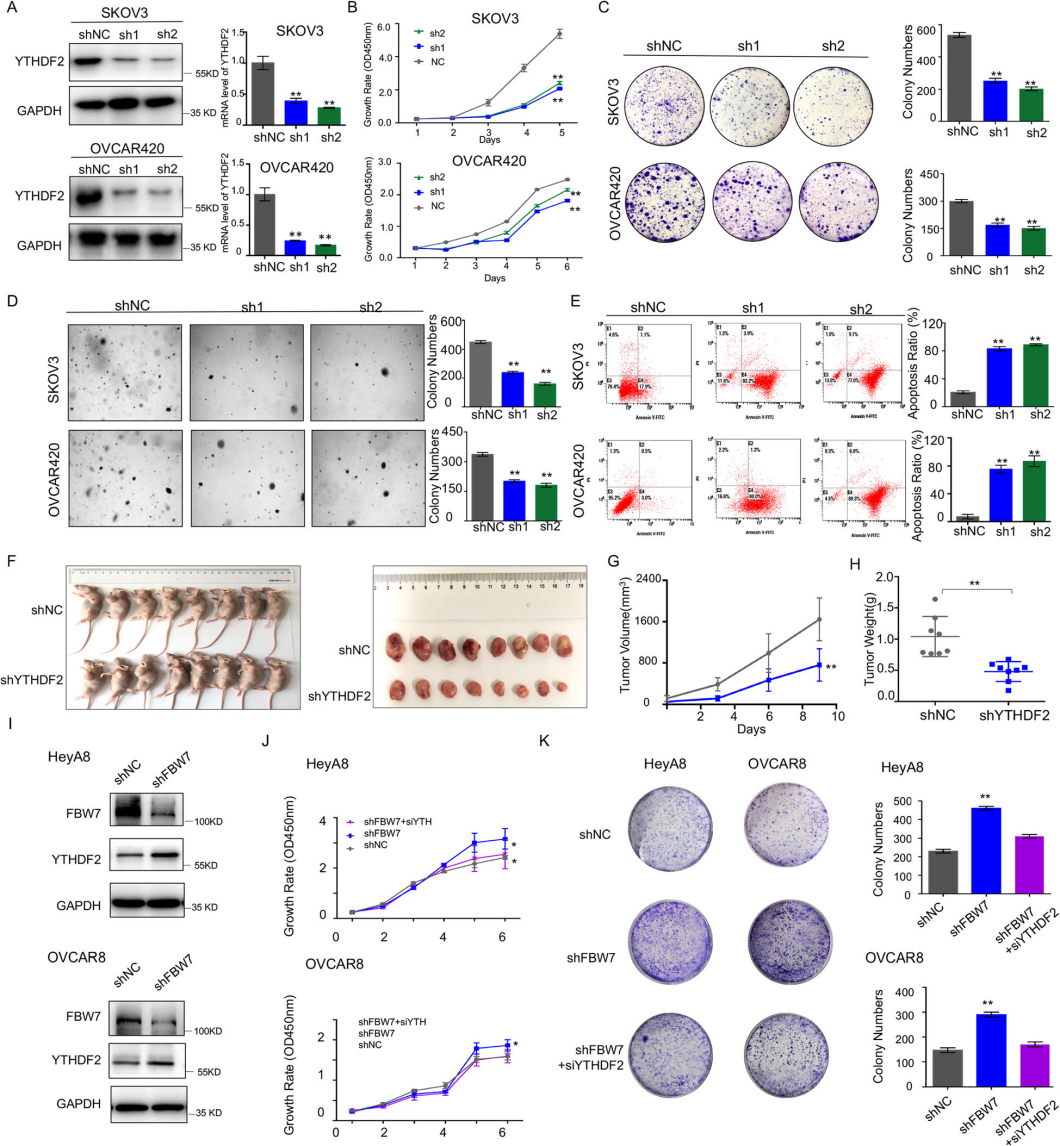
Ablation of YTHDF2 significantly suppresses ovarian cancer cell proliferation and growth in vitro and in vivo. **a** Stable expression of shRNAs against YTHDF2 in SKOV3 and OVCAR420 cells. The gene knockdown efficiency is verified at both mRNA and protein levels. **b** YTHDF2 depletion significantly reduces the proliferation rate of SKOV3 and OVCAR420 cells. **c** YTHDF2 depletion impairs the colony-forming ability of ovarian cancer cells. **d** YTHDF2 depletion suppresses anchorage-independent growth of ovarian cancer cells. **e** YTHDF2 depletion promotes ovarian cancer cell apoptosis. **f** YTHDF2 depletion in SKOV3 cells markedly suppresses ovarian tumor growth in the xenograft mice. **g** The size of tumors formed in the xenograft mice is monitored every 3 days. (H) YTHDF2 depletion significantly reduces weight of tumors in the xenograft mice. ** *P* < 0.01. Results were presented as mean ± SD of three independent experiments. *P < 0.05 or **P < 0.01 indicates a significant difference between the indicated groups (**i**, **j**, **k**).

Although YTHDF2 was found to be involved in the development of several cancers [26–29, 33], it remains largely elusive if the protein is required for ovarian cancer cell survival and growth. To test this speculation, the YTHDF2-depleted SKOV3 and OVCAR420 cell lines were generated using two independent shRNAs (Fig. 5a). We showed that ablation of YTHDF2 significantly prohibits proliferation (Fig. 5b), colony-forming ability (Fig. 5c), and anchorage-independent growth (Fig. 5d) of the two ovarian cancer cell lines. Also, the flow cytometry analysis indicated that knockdown of YTHDF2 drastically triggers ovarian cancer cell apoptosis (Fig. 5e). Moreover, the xenograft mouse model revealed that YTHDF2 depletion markedly hampers ovarian tumor growth in vivo (Fig. 5f) leading to significant reduction of the tumor volume and weight (Fig. 5g and h). And Knockdown of YTHDF2 significantly restores cell proliferation and colony-forming ability induced by FBW7 depletion (Fig. 5i-k). Thus, these findings indicate that YTHDF2 is essential for ovarian cancer cell propagation. To confirm these results, we also evaluated the biological function of ectopic YTHDF2 in ovarian cancer cell lines. By examining the expression of YTHDF2 in multiple ovarian cancer cell lines, we generated YTHDF2-stably overexpressing lineages using OVCAR433 and OVCAR8 cells that sustain relatively low expression of endogenous YTHDF2 (Supplementary Fig. 6 and 7A). As expected, overexpression of YTHDF2 significantly boosted cell proliferation (Supplementary Fig. 7B), colony-forming ability (Supplementary Fig. 7C), and anchorage-independent growth (Supplementary Fig. 7D). Collectively, we demonstrate that YTHDF2 may drive ovarian cancer development, and that FBW7 restrains ovarian carcinogenesis by inducing proteolytic degradation and thus compromising the oncogenic activity of YTHDF2.

### Identification of BMF as an effector of the FBW7-YTHDF2 cascade

**Fig. 6:**
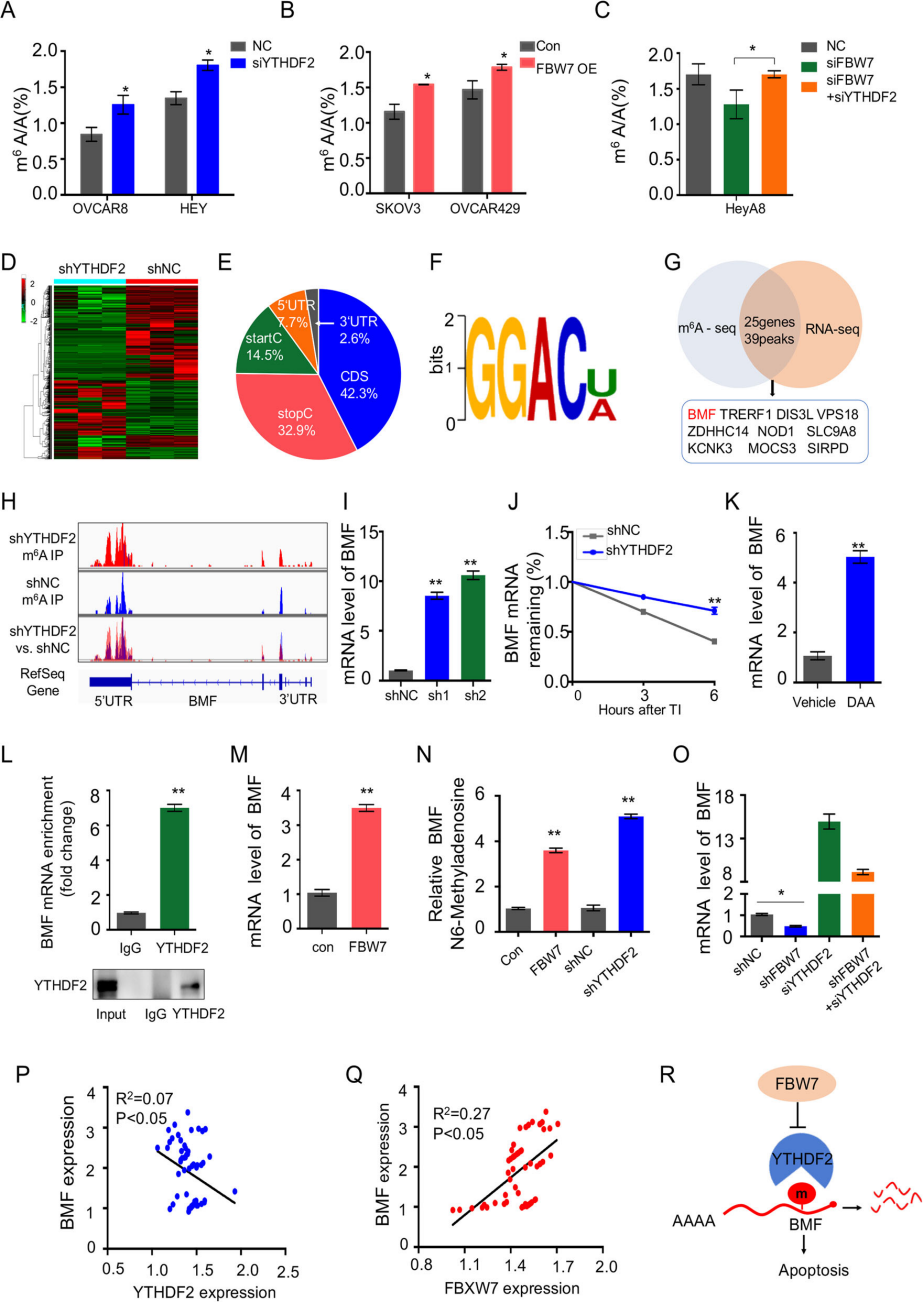
Regulation of m^6^A-modified mRNA stability by the FBW7-YTHDF2 cascade. **a** Induction of the mRNA m^6^A levels upon YTHDF2 depletion in OVCAR8and HEY cells. The RNA m^6^A quantification was conducted by LC-MS/MS. [31] The YTHDF2 depletion efficiency was shown in Supplementary Fig. 9A. **b** Induction of the mRNA m^6^A levels upon FBW7 overexpression in SKOV3 and OVCAR429 cells. The FBW7 overexpression efficiency was shown in Supplementary Fig. 9B. **c** Decline of the mRNA m^6^A level caused by FBW7 depletion can be rescued by knockdown of YTHDF2. The YTHDF2 depletion and FBW7 overexpression efficiency was shown in Supplementary Fig. 9C. **d** RNA-sequencing analysis reveals a distinct gene expression patterns in response to YTHDF2 depletion in SKOV3 cells. **e** Distribution of m^6^A modification in mRNA transcripts. The m^6^A signal is mostly enriched in the coding sequences. **f** The m^6^A consensus sequence motif is identified in ovarian cancer cells. **g** Integration of the RNA-Sequencing and m^6^A-Sequencing results revealed 25 genes with 39 peaks are consistently elevated upon YTHDF2 knockdown, which are displayed in Supplementary table 4 (**h**). The m^6^A modification site of BMF mRNA was in the 3′-UTR region. **i** Knockdown of YTHDF2 increases BMF mRNA level in SKOV3 cells. **j** Knockdown of YTHDF2 prolongs the half-life of BMF mRNA. Cells were treated with actinomycin D (5 μg/ml) for 3 or 6 h followed by RT-qPCR analysis. **k** The mRNA level of BMF is elevated upon the treatment of 50 μM 3-deazaadenosine (DAA) in SKOV3 cells. **l** The interaction between YTHDF2 and BMF pre-mRNA was detected by the RIP assay. **m** The mRNA level of BMF is significantly elevated in SKOV3 cells stably overexpressing FBW7. **n** The relative m6A level of BMF pre-mRNA was detected by MeRIP-qPCR in SKOV3 cells. **o** The FBW7-mediated regulation of BMF is dependent on YTHDF2 demonstrated by qRT-PCR detection. **p** YTHDF2 is inversely correlated with BMF expression in 64 epithelial ovarian cancer tissues. **q** FBW7 is positively correlated with BMF expression in 64 epithelial ovarian cancer tissues. **r** The working model for the regulation of m^6^A-dependent turnover of BMF mRNA by the FBW7-YTHDF2 cascade. Results were presented as mean ± SD of three independent experiments. *P < 0.05 or **P < 0.01 indicates a significant difference between the indicated groups

Since YTHDF2 was shown to prompt the decay of the m^6^A-modified transcripts [24], we determined if FBW7 regulates cellular m^6^A enrichment through inhibition of YTHDF2. By employing a LC-MS/MS assay that allows quantification of m^6^A level in cells, we showed that ablation of YTHDF2 significantly increases m^6^A abundance in the ovarian cancer cell lines, OVCAR8 and HEY (Fig. 6a), which is in accordance with a previous study performed in HeLa cells [24]. Remarkably, overexpression of FBW7 led to significant elevation of m^6^A level in SKOV3 and OVCAR429 cell lines (Fig. 6b), which phenocopies the effect of YTHDF2 depletion. In addition, we assessed m^6^A enrichment in ovarian cancer tissues with high or low expression level of FBW7, which again verified that FBW7 expression is positively associated with m^6^A level in tumors (Fig. 1g). Of note, we also ascertained that FBW7-mediated regulation of m^6^A abundance is dependent on YTHDF2, as evidenced by the fact that knockdown of YTHDF2 dramatically neutralized the effect of FBW7 depletion on m^6^A level (Fig. 6c).

Next, we examined if YTHDF2 orchestrates gene expression in ovarian cancer by conducting an RNA-sequencing analysis (Fig. 6d). Ablation of YTHDF2 led to upregulation of 348 genes and downregulation of 320 genes in SKOV3 cells. We also performed the genome-wide mapping of the m^6^A modification in control and YTHDF2-depleted SKOV3 cell lines through a MeRIP (m^6^A)-sequencing assay. The results indicated that the m^6^A peaks mainly locate in the CDS regions (42.3%), then the stop codon sites (32.9%), start codon sites (14.5%), 5′ UTRs (7.7%) and 3′ UTRs (2.6%) (Fig. 6e). The m^6^A consensus sequence identified in SKOV3 cells was GGAC [U/A] (Fig. 6f) that is consistent with previous studies [24, 34], indicating that YTHDF2 may also bind to m^6^A in ovarian cancer. By integrating the RNA-Sequencing and m^6^A-Sequencing results, we found that 25 genes with 39 peaks are consistently elevated upon YTHDF2 knockdown (Fig. 6g and Supplementary table 4). Among these dysregulated genes, *BMF* was considered an interesting target given that it encodes a pro-apoptotic BH3-only Bcl2 family protein required for inhibiting pro-survival Bcl2 members and triggering apoptosis [35] and that ablation of YTHDF2 exhibited higher peak enrichment compared to the control group (Fig. 6h). We further testified that ablation of YTHDF2 significantly increases BMF mRNA expression level (Fig. 6i) and prolongs its half-life (Fig. 6j), which validates BMF mRNA as a target of YTHDF2 for degradation. Since the m^6^A modification is indispensable for YTHDF2-induced mRNA decay, we determined if inhibition of m^6^A stabilizes BMF mRNA. As shown in Fig. 6k, treatment of SKOV3 cells with the global methylation inhibitor, 3-deazaadenosine (DAA), significantly elevated the mRNA level of BMF, suggesting that methylation is critical for degradation of BMF mRNA (Fig. 6k). The interaction between YTHDF2 and BMF was verified by the RIP assay (Fig. 6l). And overexpression of YTHDF2 significantly reduces the expression of BMF (Supplementary Fig. 8A). The effect of YTHDF2 on cell proliferation and apoptosis can be reversed by the restoration of BMF expression (Supplementary Fig. 8B-E). Moreover, overexpression of FBW7, like knockdown of YTHDF2, strikingly induced BMF mRNA level (Fig. 6m), which is owing to YTHDF2 degradation by ectopic FBW7 (Fig. 3c-f). Further, the overexpression of FBW7 or knockdown of YTHDF2 increased the levels of m6A modification of BMF pre-mRNA respectively, compared with modification levels of control vectors in SKOV3 cells (Fig. 6n).

We also showed that the FBW7-mediated regulation of BMF is dependent of YTHDF2 (Fig. 6o and Supplementary Fig. 12,). Importantly and in line with these in vitro results, we also revealed that the expression of BMF is inversely correlated with YTHDF2 expression (Fig. 6p), whereas is positively associated with FBW7 expression (Fig. 6q, Supplementary Fig. 10), in ovarian cancer tissues. And the mRNA levels of BMF were elevated in patients with tumors expressing higher levels of FBW7, while YTHDF2 mRNA levels demonstrate no significant difference (Supplementary Fig. 11). Collectively, our results clearly demonstrate that FBW7 provokes the expression of pro-apoptotic BMF through an m^6^A-dependent mechanism by interacting with and degrading the m^6^A reader YTHDF2 in ovarian cancer (Fig. 6r).

## Discussion

The tumor suppressor FBW7 maintains genomic stability and prevents tumor growth by degrading multiple oncoproteins in a variety of human cancers. Here, we have unveiled that the m^6^A-binding protein YTHDF2 acts as a tumor promoter bolstering ovarian cancer cell propagation and the proteolytic degradation of YTHDF2 induced by FBW7 is required for the tumor inhibitory activity of the latter. Mechanistically, FBW7 enhances stabilization of the pro-apoptotic BMF mRNA by abating YTHDF2-mediated m^6^A-dependent mRNA decay. Therefore, our study depicts the role of the FBW7-YTHDF2-BMF cascade in the development of ovarian cancer.

Since a seminal study suggested that FBW7, the human homologue of *Drosophila* archipelago, may be involved in ovarian carcinogenesis by regulating Cyclin E expression [36], the expression pattern, mutation frequency, and clinical relevance of FBW7 in ovarian cancer has been evaluated [19–21]. In the present study, we have revealed that FBW7 is downregulated in ovarian carcinomas compared to non-cancerous ovarian tissues (Fig. 1a-b, Supplementary Fig. 1A-B) and that lower expression level of FBW7 is associated with unfavorable prognosis (Fig. 1e-f, Supplementary Fig. 1C), which is consistent with the previous study [21]. These observations were further attested on the grounds of the fact that ectopic FBW7 impedes, while ablation of FBW7 fosters, ovarian cancer cell survival and growth (Fig. 2, Supplementary Fig. 3). It was found that FBW7 prompts ubiquitination and degradation of MCL1, while lack of FBW7 elevates MCL1 expression leading to resistance to antitubulin therapy in ovarian cancer [37]. Also, Melanoma-associated antigen A1 (MAGEA1) was shown to suppress cell proliferation and migration probably through FBW7-mediated NICD1 degradation in ovarian cancer [38]. These findings definitely underscored the importance of FBW7 in ovarian cancer. Although a few substrates of FBW7 has been reported as mentioned above, our study uncovers the m^6^A binding protein YTHDF2 as an additional substrate of FBW7 through a co-IP-MS analysis (Fig. 3a-b, Supplementary Fig. 4), and elaborates that FBW7 enhances ubiquitination and proteolytic degradation of YTHDF2 (Fig. 3c-f), which presents a novel mechanism behind FBW7-mediated tumor suppression.

YTHDF2 is an m^6^A reader that promotes rapid decay of m^6^A-modified transcripts [24]. Several studies indicate that YTHDF2 can perform either as an oncogenic protein or as a tumor suppressor relying on the context of different cancers [26–29] or by regulating different mRNA targets [39]. Remarkably, we have provided the first line of evidence demonstrating that YTHDF2 is a tumor promoter in ovarian cancer, because ablation of YTHDF2 dampens cell proliferation and enhances apoptosis (Fig. 5), which recapitulates the tumor phenotype in vitro and in vivo caused by FBW7 overexpression (Fig. 2). The role of YTHDF2 in propelling ovarian cancer development is also verified by the clinical observation that YTHDF2 is upregulated in ovarian cancer samples and negatively associated with prognosis (Fig. 4a, e, Supplementary Fig. 5). Together with the findings that FBW7 induces YTHDF2 degradation in ovarian cancer cells (Fig. 3c-f) and inversely correlates with the expression of the latter in ovarian cancer tissues (Fig. 4b-c), we have convincingly demonstrated that FBW7 suppresses ovarian carcinogenesis and progression by antagonizing the oncogenic activity of YTHDF2.

It has been shown that YTHDF2 targets a plethora of mRNAs for degradation in cancers, such as, TNFR2, c-Myc and CEBPA in leukemia [26, 27], EGFR, IL11, SERPINE2 and SOCS2 in liver cancer [28, 29, 39], and PD-1, CXCR4, and SOX10 in melanoma [33]. In our attempt to identify YTHDF2-targeting mRNAs in ovarian cancer, the RNA-sequencing and m^6^A-sequencing analyses were conducted, leading to the identification of a number of genes that could be regulated by YTHDF2 in an m^6^A-dependent manner (Fig. 6g and Supplementary table 7). The pro-apoptotic BMF was further investigated as a key effector of the FBW7-YTHDF2 cascade in ovarian cancer, because BMF was found to play critical roles in restraining malignancies [40] and maintaining the restricted number of primordial follicles and germ cells in the ovary by triggering apoptosis [41]. Through the m^6^A-sequencing experiment, we found that knockdown of YTHDF2 increases the m^6^A accumulation in the 5′ UTR of BMF (Fig. 6h), suggesting that YTHDF2 depletion may elevate the level of this m^6^A-modified mRNA. Indeed, our further results indicate that knockdown of YTHDF2 increases the expression of BMF by enhancing the stability of its mRNA (Fig. 6j-k). Most importantly, FBW7 elevates BMF mRNA level and is positively associated with BMF expression in primary ovarian cancer tissues (Fig. 6q). These results mechanistically support the biological function of the FBW7-YTHDF2 cascade in triggering apoptosis and inhibiting growth of ovarian carcinoma. In addition, several other potentially interesting targets of YTHDF2 in ovarian cancer have been identified, such as the putative tumor suppressors TRERF1, ZDHHC14, DIS3L, Vps18, NOD1, and SLC9A8. These proteins were found to be involved in the regulation of cell cycle arrest [42], caspase-dependent apoptosis [43], microRNA stability [44], ER signaling pathway [45], and inflammation-associated tumorigenesis [46, 47], respectively. Altogether, these findings profoundly demonstrate the role of the FBW7-YTHDF2 axis in restricting ovarian cancer by derepressing various tumor suppressors.

Given that ovarian cancer sustains the highest frequency of *TP53* gene mutation which leads to the downregulation of FBW7 [6, 7, 21], our study indicates a potential interplay between mutant p53 and the FBW7-YTHDF2 axis, and suggests an alternative working model for the “gain-of-function” of mutant p53 through the regulation of m^6^A-dependent RNA turnover [48]. Interestingly, a recent study showed that mutant p53-R273H impairs BMF mRNA expression in breast and colon cancer cell lines [49], which is in agreement with and could be explained by our proposed model. Hence, it is worthwhile investigating in future if mutant p53 controls gene expression via regulation of the FBW7-YTHDF2 axis in ovarian cancer.

## Conclusions

In conclusion, our study explicitly demonstrates an unappreciated FBW7-YTHDF2-BMF axis in ovarian cancer. FBW7 interacts with and degrades the oncogenic YTHDF2, consequently leading to stabilization of m^6^A-modified mRNAs, including the pro-apoptotic gene BMF, and impairment of ovarian cancer cell survival and proliferation. Our findings also unveil the clinical significance of the axis, which would be informative for future development of anticancer therapies.

## Supplementary Information

**Additional file 1: Figure S1.** (A) Expression of FBW7 in cancerous and normal ovarian tissues detected by IB, which is a supplement to Fig. 1a. (B) FBW7 is downregulated in the TCGA ovarian cancer cohort. (C) High expression level of FBW7 is significantly associated with better overall survival (OS) in the TCGA ovarian cancer cohort.

**Additional file 2: Figure S2.** FBW7 expression in various human ovarian cancer cell lines.

**Additional file 3: Figure S3.** Knockdown of FBW7 significantly promotes proliferation, colony formation, and anchorage-independent growth of ovarian cancer cells. (A) Stable expression of shRNAs against FBW7 in HeyA8 and OVCAR8 cells. The gene knockdown efficiency is validated at both mRNA and protein levels. (B) Knockdown of FBW7 significantly increases the proliferation rate of HeyA8 and OVCAR8 cells. (C) Knockdown of FBW7 enhances colony-forming ability of ovarian cancer cells. (D) Knockdown of FBW7 boosts anchorage-independent growth of ovarian cancer cells. Results were presented as mean ± SD of three independent experiments. *P < 0.05 or **P < 0.01 indicates a significant difference between the indicated groups.

**Additional file 4: Figure S4.** (A-B) Validation of FBW7 accumulation in the immunoprecipitates by IB (A) and silver staining (B). (C) Mass spectrometry analysis of the immunoprecipitates with IgG or the anti-FBW7 antibody. Results were presented as mean ± SD of three independent experiments.

**Additional file 5: Figure S5.** (A) YTHDF2 is upregulated in the TCGA ovarian cancer cohort. (B) High expression level of YTHDF2 is significantly associated with poor overall survival (OS) in the TCGA ovarian cancer cohort.

**Additional file 6: Figure S6.** YTHDF2 expression in various human ovarian cancer cell lines.

**Additional file 7: Figure S7.** Overexpression of YTHDF2 significantly promotes proliferation, colony formation, and anchorage-independent growth of ovarian cancer cells. (A) Stable overexpression of YTHDF2 in OVCA433 and OVCAR8 cells. The expression of YTHDF2 is validated at both mRNA and protein levels. (B) Overexpression of YTHDF2 significantly increases the proliferation rate of OVCA433 and OVCAR8 cells. (C) Overexpression of YTHDF2 enhances colony-forming ability of ovarian cancer cells. (D) Overexpression of YTHDF2 boosts anchorage-independent growth of ovarian cancer cells. Results were presented as mean ± SD of three independent experiments. *P < 0.05 or **P < 0.01 indicates a significant difference between the indicated groups.

**Additional file 8: Figure S8.** (A) Overexpression of YTHDF2 significantly reduces the expression of BMF. (B) The effect of YTHDF2 on cell proliferation and colony-forming ability can be reversed by the restoration of BMF expression. (C) The BMF overexpression in SKOV3 and OVCAR429 cells increases apoptosis; (D) The YTHDF2 overexpression in the FBW7 OE stable cells attenuates the apoptosis caused by FBW7 overexpression; (E) Knockdown of BMF in the YTHDF2 shRNA-stable cells reduced the apoptosis caused by the knockdown of BMF.

**Additional file 9: Figure S9.** Efficiency of gene knockdown and overexpression was validated. (A) Knockdown of YTHDF2 was validated in OVCAR8 and HEY cells. (B) Overexpression of FBW7 was validated in SKOV3 and OVCAR429 cells. (C) Knockdown of FBW7 and YTHDF2 was validated in OVCAR8 cells.

**Additional file 10: Figure S10.** IHC staining of BMF in human the ovarian cancer tissue and normal ovary tissue.

**Additional file 11: Figure S11.** The YTHDF2 and BMF mRNA level in FBW7 high and low group.

**Additional file 12: Figure S12.** The FBW7-mediated regulation of BMF is dependent on YTHDF2 demonstrated by WB detection.

**Additional file 13: Figure S13.** (A) The OS curves of patients with different expression levels of FBW7 and YTHDF2. (B) The PFS curves of patients with different expression levels of FBW7 and YTHDF2. OS, overall survival; PFS, progress free survival.

**Additional file 14: Table S1.** shRNA sequences and primer sequences for RT-qPCR.

**Additional file 15: Table S2.** The relationship between FBW7 expression and the clinicopathological features of EOC.

**Additional file 16: Table S3.** The list of differentially identified proteins by the co-IP-mass spectrometry analysis.

**Additional file 17: Table S4.** The relationship between YTHDF2 expression and the clinicopathological features of EOC.

**Additional file 18: Table S5.** Correlations between the expression of FBW7/YTHDF2 and clinicopathological characteristics.

**Additional file 19: Table S6.** Multivariate analysis of variables associated with PFS and OS.

**Additional file 20: Table S7.** The list of 25 genes identified by integrating the RNA-sequencing and m6A-sequencing results.

## Data Availability

Supplementary Table S1, S2, S3, S4, S5, S6 and S7 and Figs. S1, S2, S3, S4, S5, S6, S7, S8, S9, S10, S11, S12 and S13 are attached.

## Abbreviations

FBW7: F-box and WD repeat domain-containing 7
YTHDF2: The human YTH domain family 2
BMF: Bcl-2 modifying factor
m^6^A: N6-methyladenosine
EOC: Epithelial ovarian cancer
SCF: Skp1-Cullin-F-box
NHEJ: Nonhomologous end-joining
UTR: Untranslated region
