# Supplementary figures and images for: FBW7 suppresses ovarian cancer development by targeting the N6-methyladenosine binding protein YTHDF2
Source: Mol Cancer. 2021 Mar 3;20:45. doi: 10.1186/s12943-021-01340-8 (PMC7927415; doi:10.1186/s12943-021-01340-8)

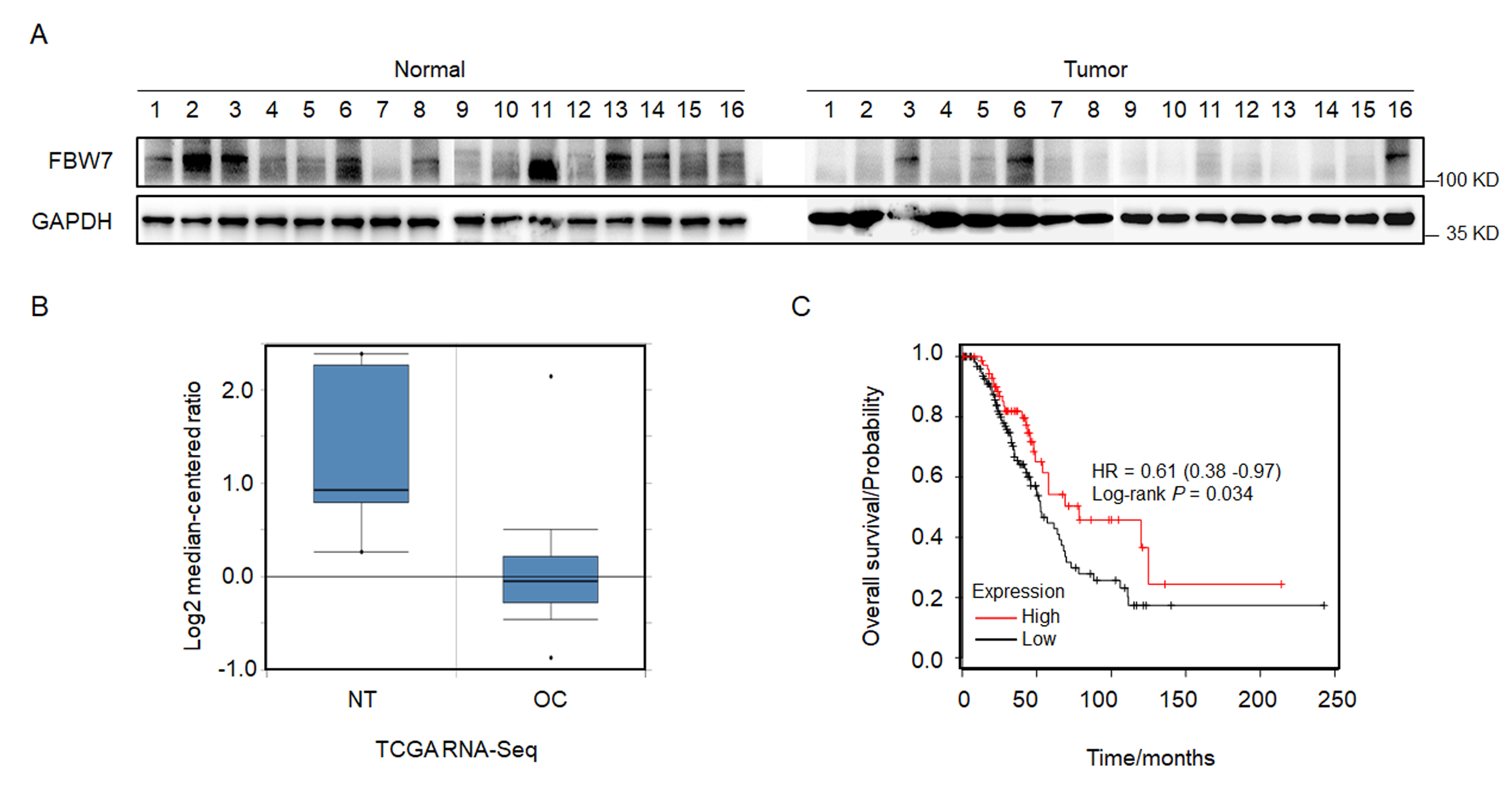

Supplement: Supplementary file 1 — Additional file 1: Figure S1. (A) Expression of FBW7 in cancerous and normal ovarian tissues detected by IB, which is a supplement to Fig. 1a. (B) FBW7 is downregulated in the TCGA ovarian cancer cohort. (C) High expression level of FBW7 is significantly associated with better overall survival (OS) in the TCGA ovarian cancer cohort. [file 12943_2021_1340_MOESM1_ESM.tif]

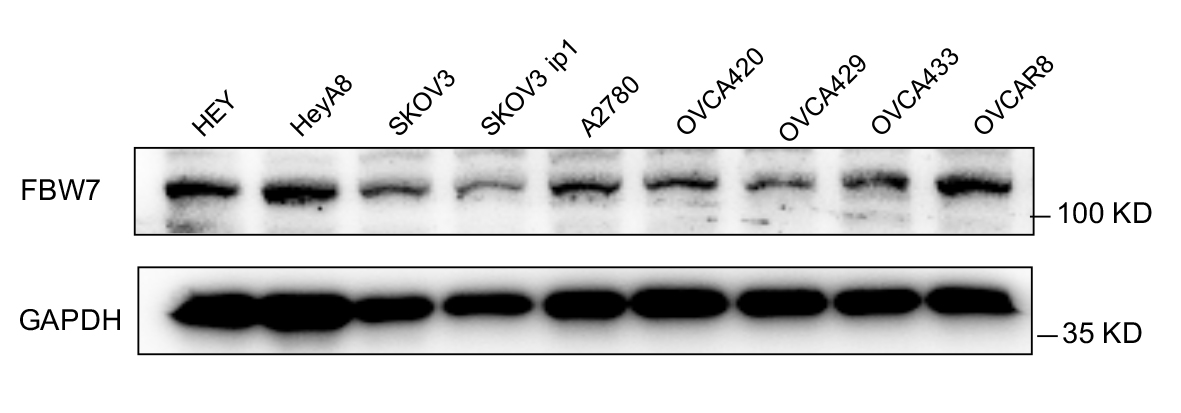

Supplement: Supplementary file 2 — Additional file 2: Figure S2. FBW7 expression in various human ovarian cancer cell lines. [file 12943_2021_1340_MOESM2_ESM.jpg]

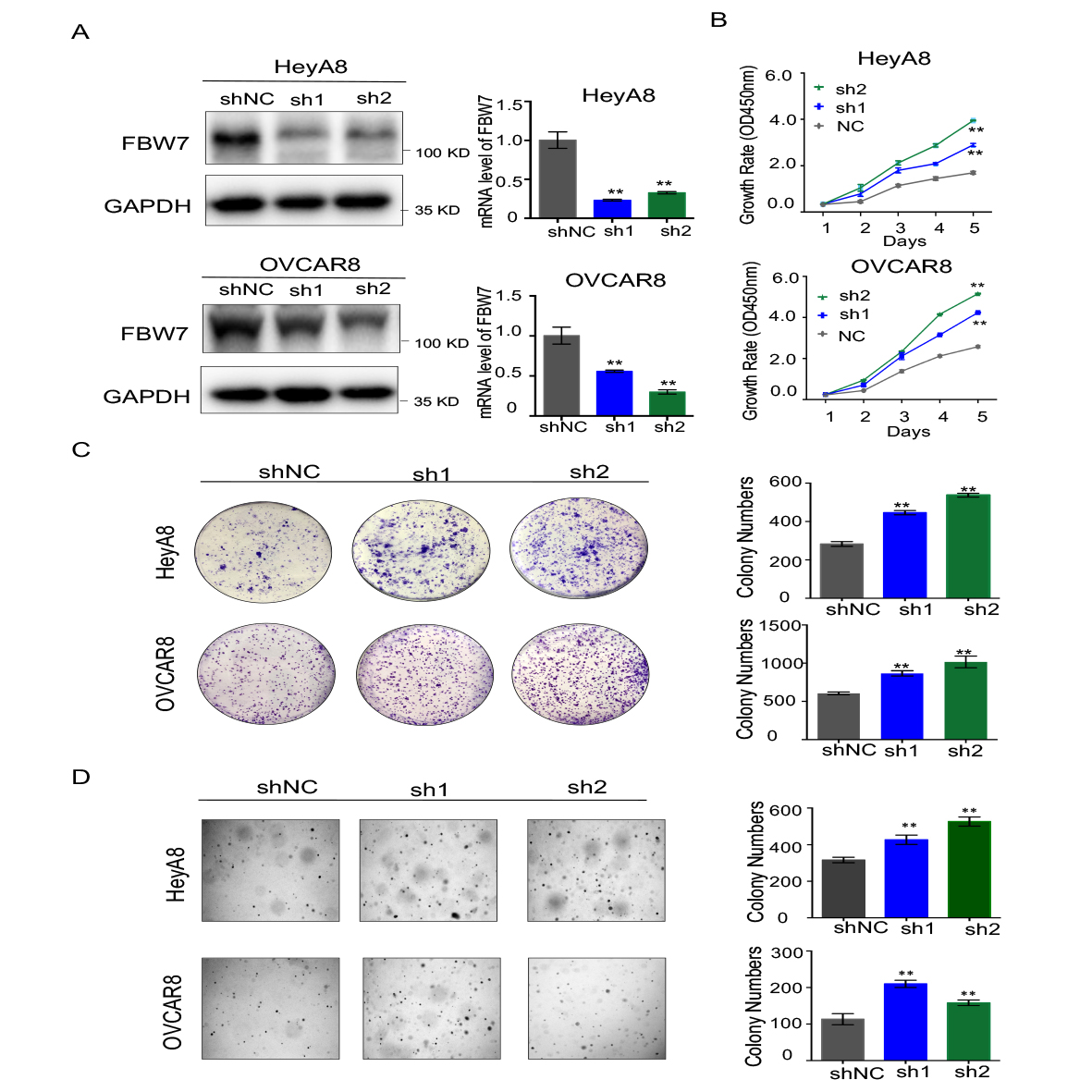

Supplement: Supplementary file 3 — Additional file 3: Figure S3. Knockdown of FBW7 significantly promotes proliferation, colony formation, and anchorage-independent growth of ovarian cancer cells. (A) Stable expression of shRNAs against FBW7 in HeyA8 and OVCAR8 cells. The gene knockdown efficiency is validated at both mRNA and protein levels. (B) Knockdown of FBW7 significantly increases the proliferation rate of HeyA8 and OVCAR8 cells. (C) Knockdown of FBW7 enhances colony-forming ability of ovarian cancer cells. (D) Knockdown of FBW7 boosts anchorage-independent growth of ovarian cancer cells. Results were presented as mean ± SD of three independent experiments. *P < 0.05 or **P < 0.01 indicates a significant difference between the indicated groups. [file 12943_2021_1340_MOESM3_ESM.jpg]

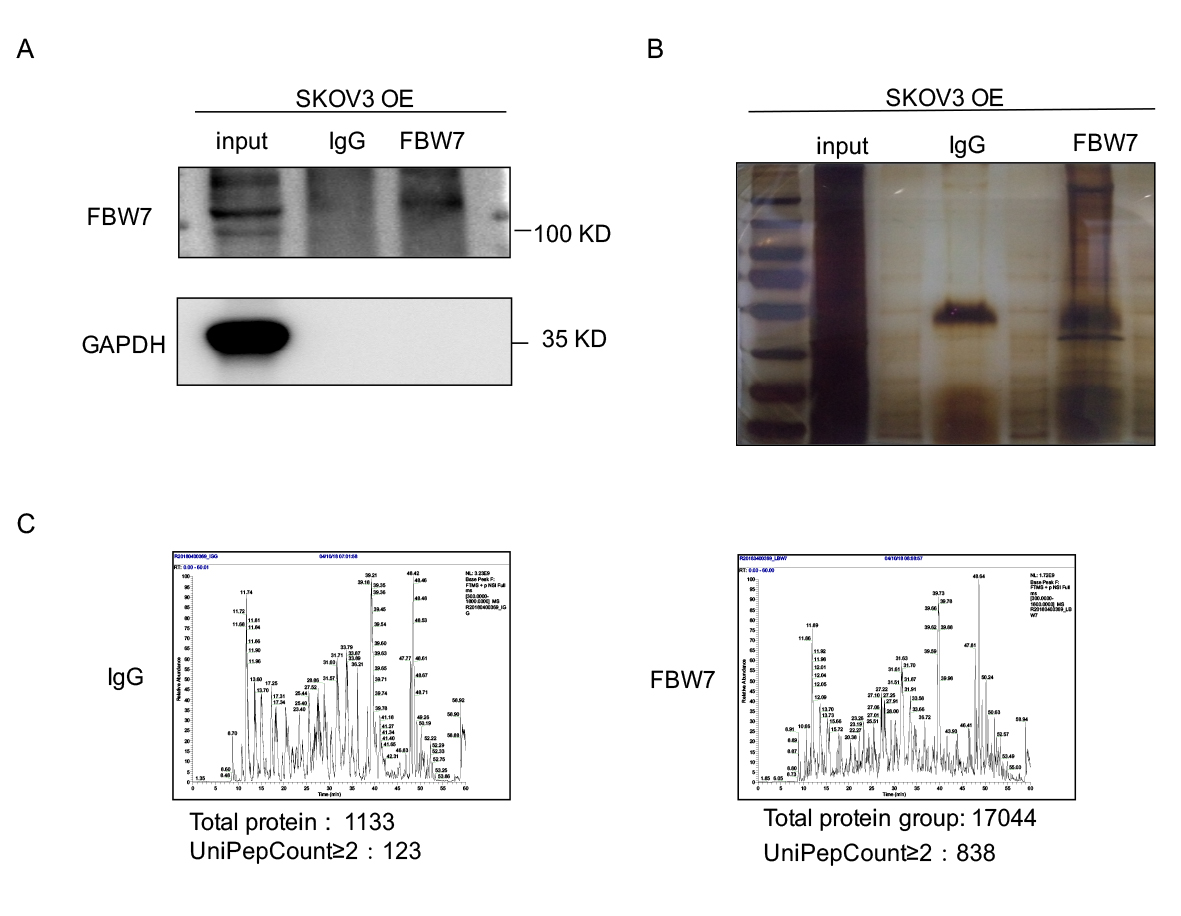

Supplement: Supplementary file 4 — Additional file 4: Figure S4. (A-B) Validation of FBW7 accumulation in the immunoprecipitates by IB (A) and silver staining (B). (C) Mass spectrometry analysis of the immunoprecipitates with IgG or the anti-FBW7 antibody. Results were presented as mean ± SD of three independent experiments. [file 12943_2021_1340_MOESM4_ESM.jpg]

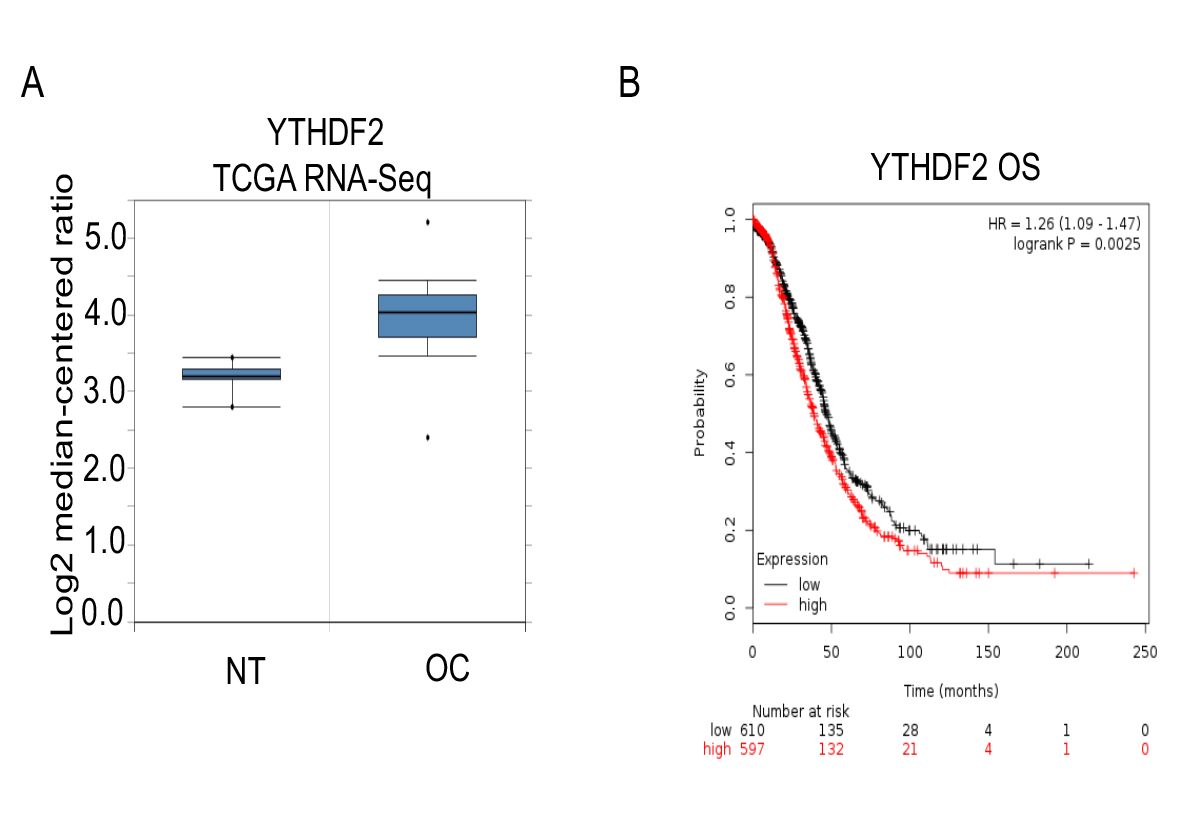

Supplement: Supplementary file 5 — Additional file 5: Figure S5. (A) YTHDF2 is upregulated in the TCGA ovarian cancer cohort. (B) High expression level of YTHDF2 is significantly associated with poor overall survival (OS) in the TCGA ovarian cancer cohort. [file 12943_2021_1340_MOESM5_ESM.jpg]

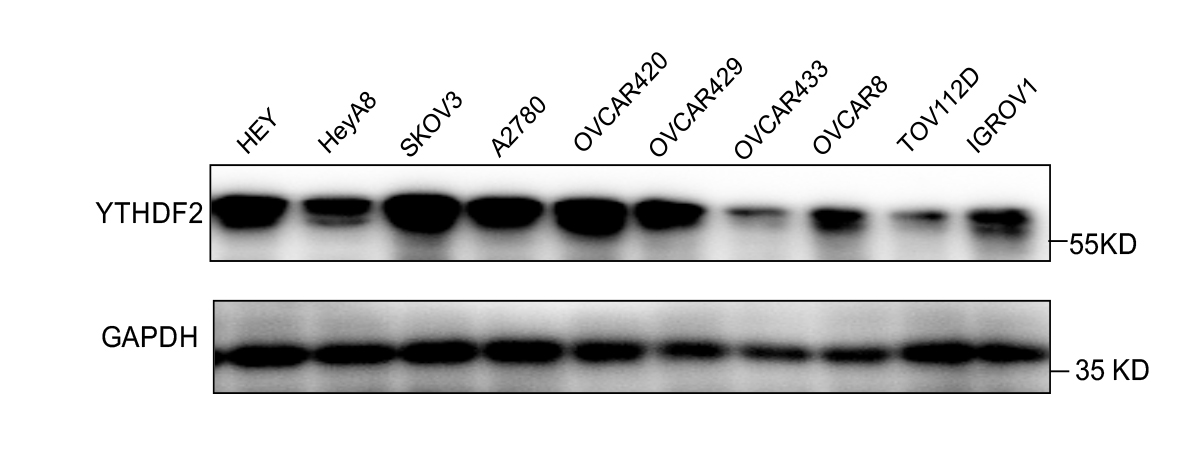

Supplement: Supplementary file 6 — Additional file 6: Figure S6. YTHDF2 expression in various human ovarian cancer cell lines. [file 12943_2021_1340_MOESM6_ESM.jpg]

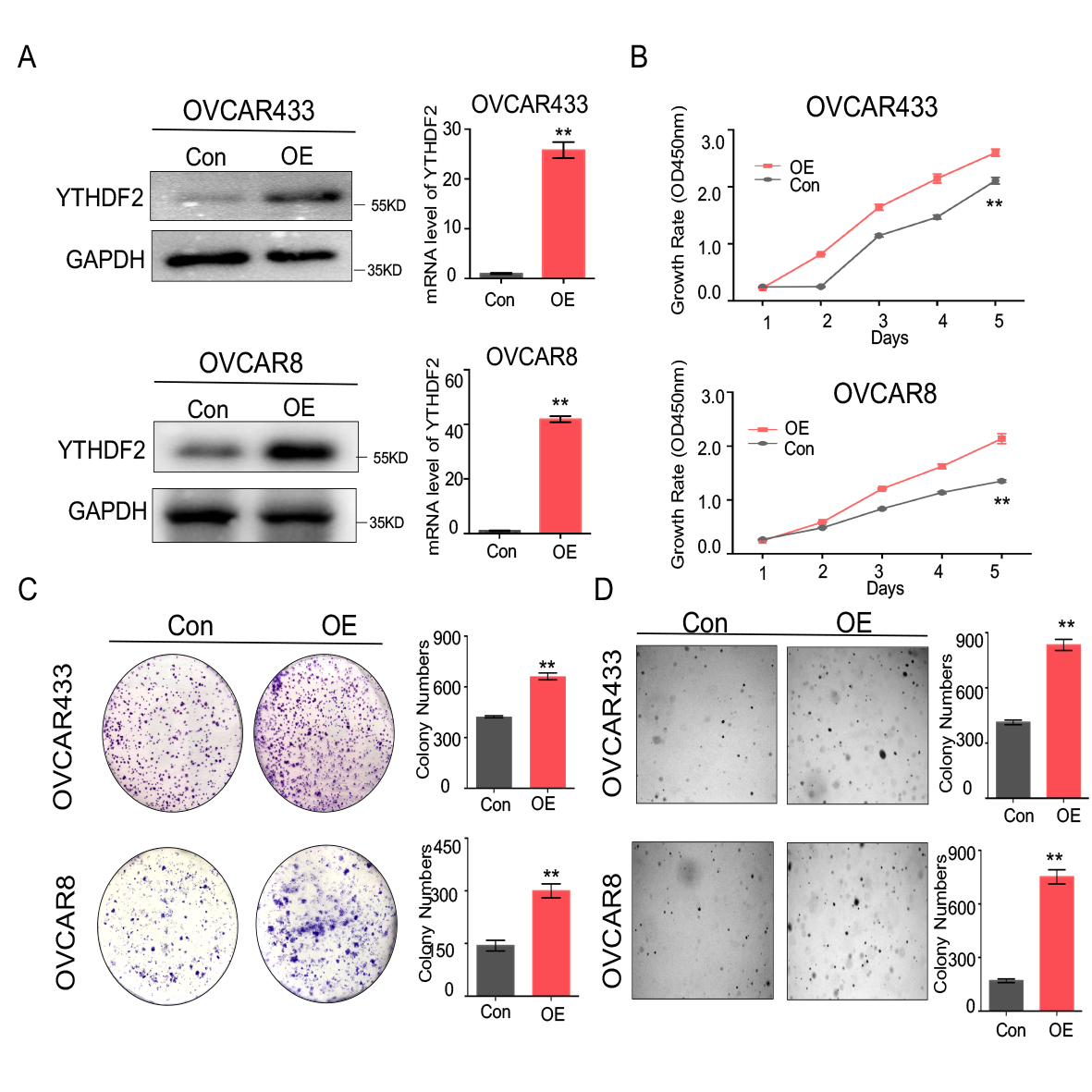

Supplement: Supplementary file 7 — Additional file 7: Figure S7. Overexpression of YTHDF2 significantly promotes proliferation, colony formation, and anchorage-independent growth of ovarian cancer cells. (A) Stable overexpression of YTHDF2 in OVCA433 and OVCAR8 cells. The expression of YTHDF2 is validated at both mRNA and protein levels. (B) Overexpression of YTHDF2 significantly increases the proliferation rate of OVCA433 and OVCAR8 cells. (C) Overexpression of YTHDF2 enhances colony-forming ability of ovarian cancer cells. (D) Overexpression of YTHDF2 boosts anchorage-independent growth of ovarian cancer cells. Results were presented as mean ± SD of three independent experiments. *P < 0.05 or **P < 0.01 indicates a significant difference between the indicated groups. [file 12943_2021_1340_MOESM7_ESM.jpg]

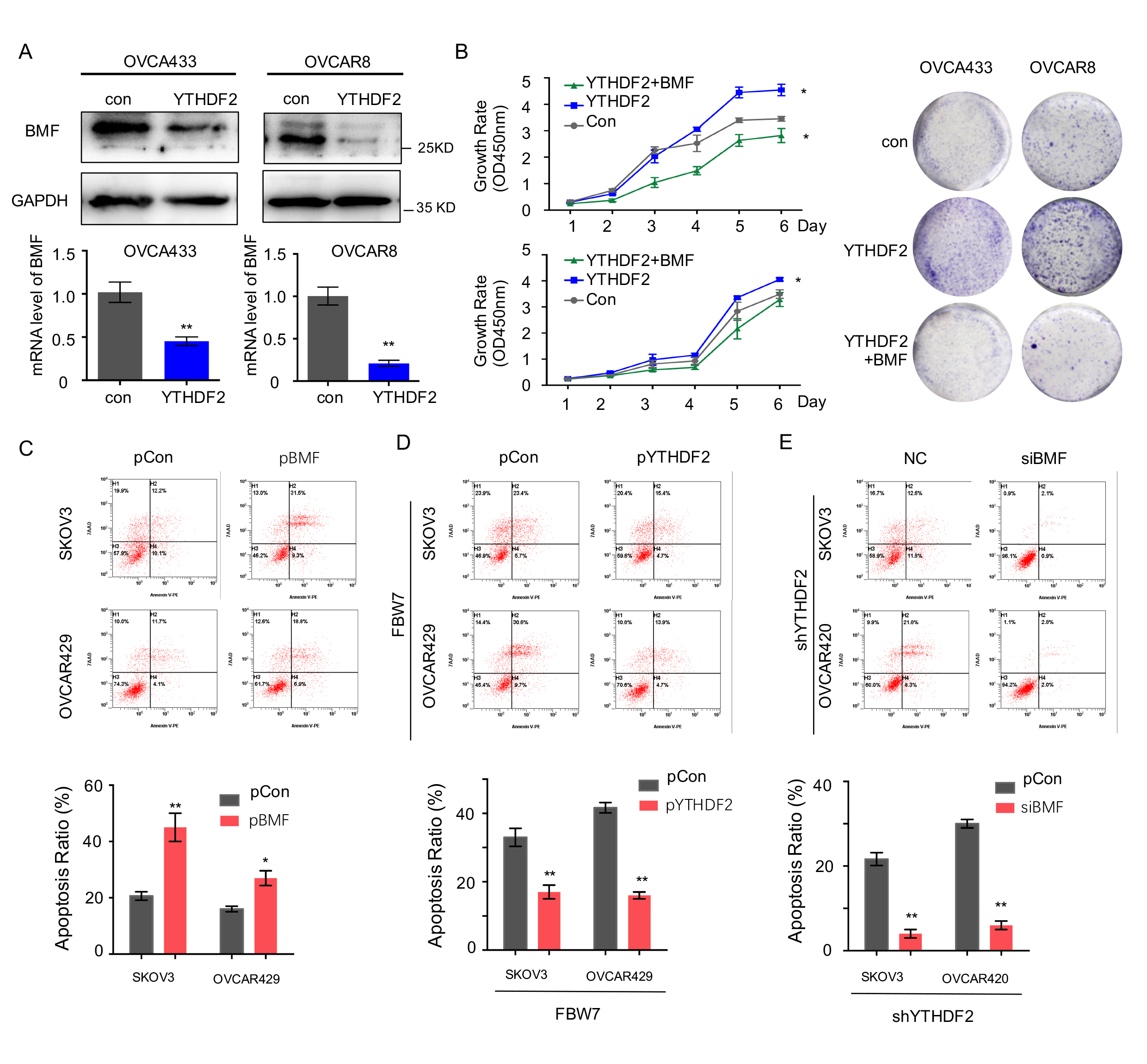

Supplement: Supplementary file 8 — Additional file 8: Figure S8. (A) Overexpression of YTHDF2 significantly reduces the expression of BMF. (B) The effect of YTHDF2 on cell proliferation and colony-forming ability can be reversed by the restoration of BMF expression. (C) The BMF overexpression in SKOV3 and OVCAR429 cells increases apoptosis; (D) The YTHDF2 overexpression in the FBW7 OE stable cells attenuates the apoptosis caused by FBW7 overexpression; (E) Knockdown of BMF in the YTHDF2 shRNA-stable cells reduced the apoptosis caused by the knockdown of BMF. [file 12943_2021_1340_MOESM8_ESM.tif]

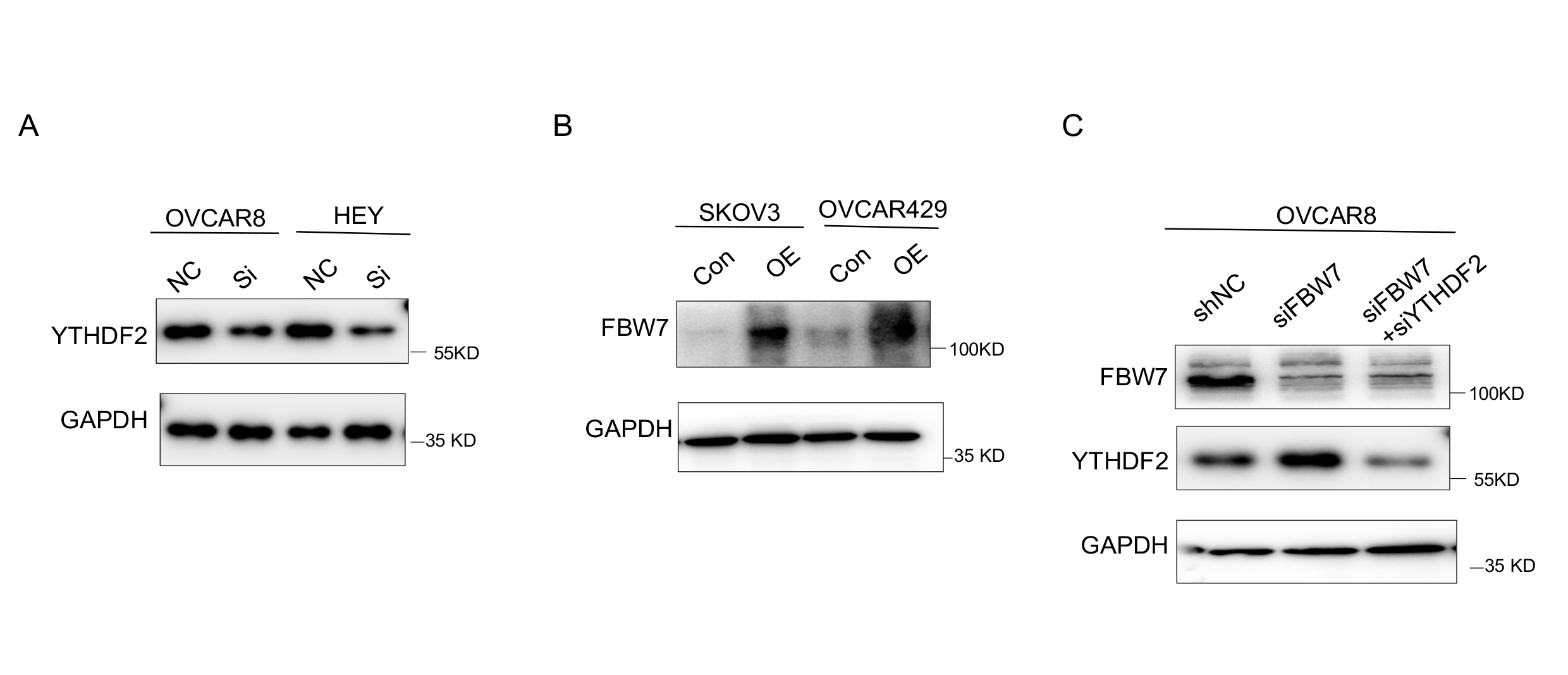

Supplement: Supplementary file 9 — Additional file 9: Figure S9. Efficiency of gene knockdown and overexpression was validated. (A) Knockdown of YTHDF2 was validated in OVCAR8 and HEY cells. (B) Overexpression of FBW7 was validated in SKOV3 and OVCAR429 cells. (C) Knockdown of FBW7 and YTHDF2 was validated in OVCAR8 cells. [file 12943_2021_1340_MOESM9_ESM.tif]

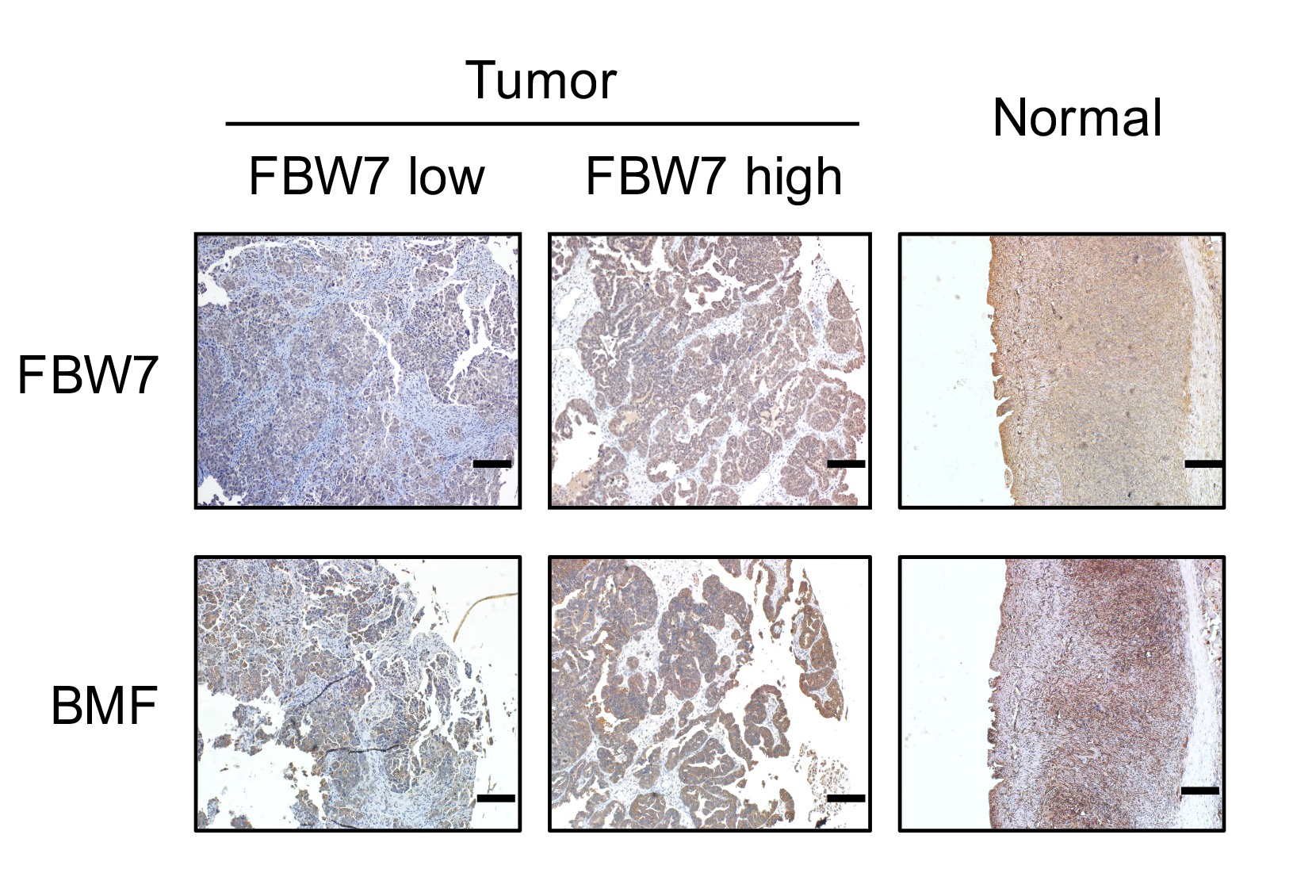

Supplement: Supplementary file 10 — Additional file 10: Figure S10. IHC staining of BMF in human the ovarian cancer tissue and normal ovary tissue. [file 12943_2021_1340_MOESM10_ESM.tif]

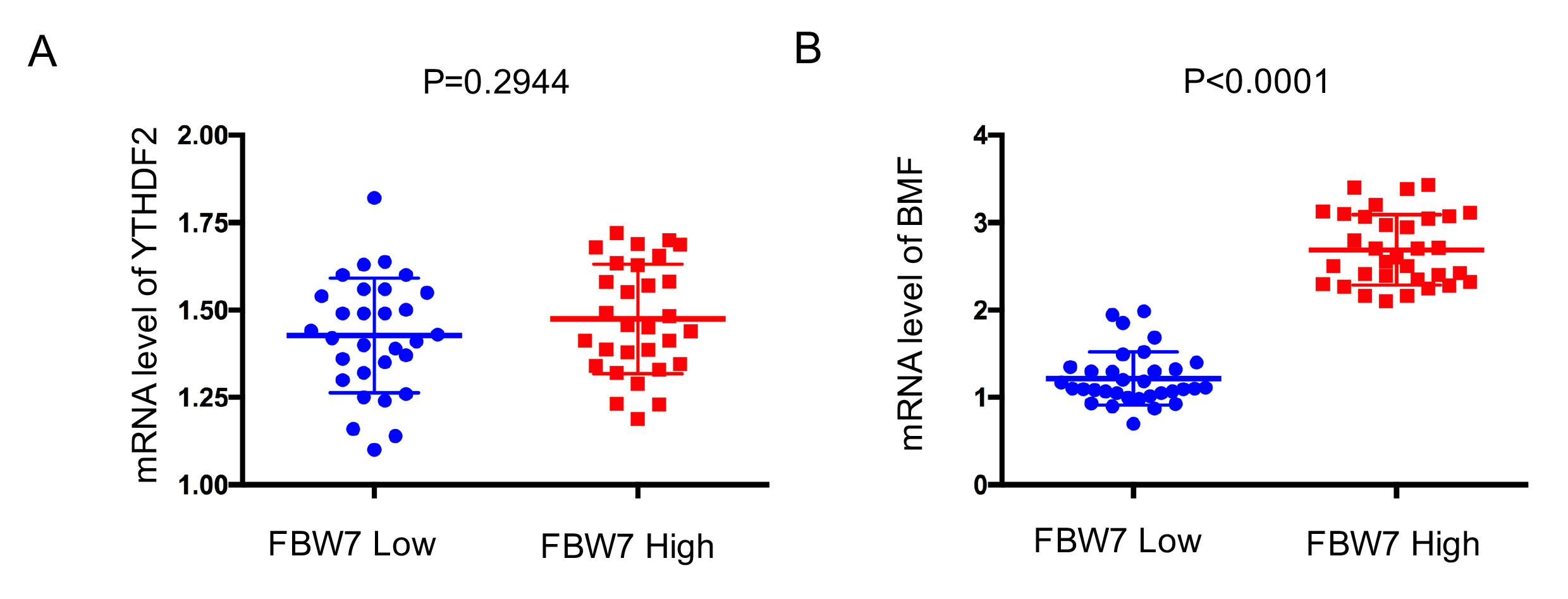

Supplement: Supplementary file 11 — Additional file 11: Figure S11. The YTHDF2 and BMF mRNA level in FBW7 high and low group. [file 12943_2021_1340_MOESM11_ESM.jpg]

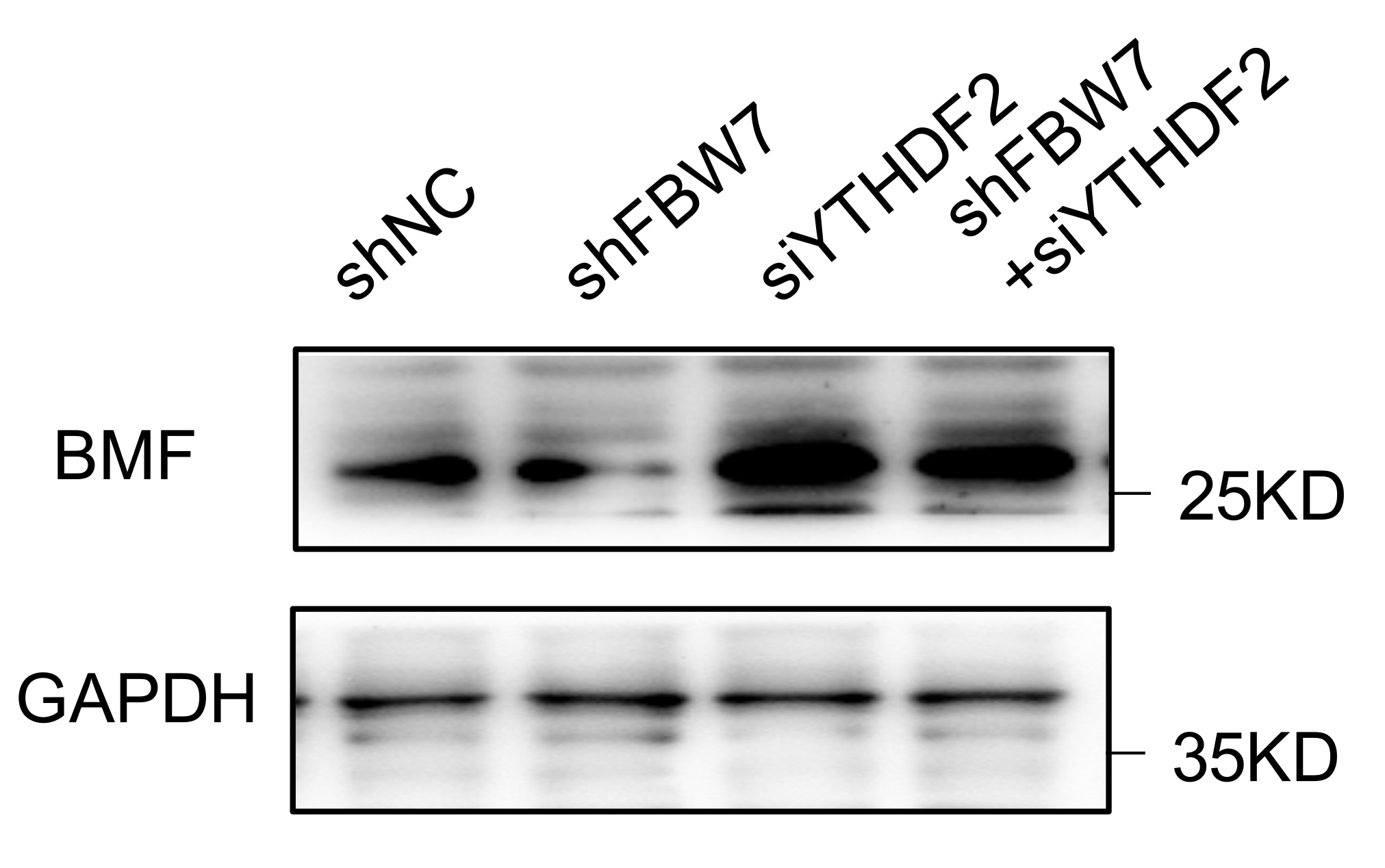

Supplement: Supplementary file 12 — Additional file 12: Figure S12. The FBW7-mediated regulation of BMF is dependent on YTHDF2 demonstrated by WB detection. [file 12943_2021_1340_MOESM12_ESM.jpg]

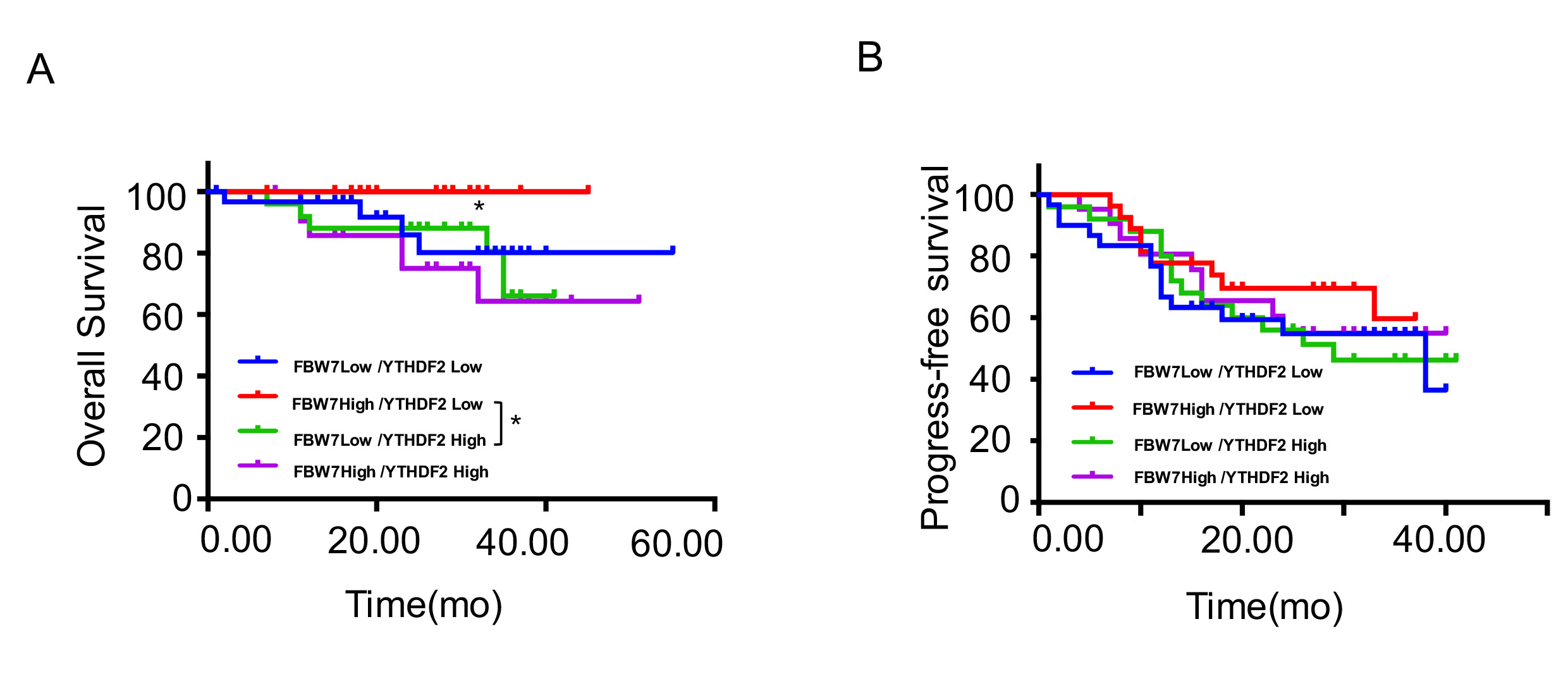

Supplement: Supplementary file 13 — Additional file 13: Figure S13. (A) The OS curves of patients with different expression levels of FBW7 and YTHDF2. (B) The PFS curves of patients with different expression levels of FBW7 and YTHDF2. OS, overall survival; PFS, progress free survival. [file 12943_2021_1340_MOESM13_ESM.jpg]
