# Supplementary material for: FBW7 suppresses ovarian cancer development by targeting the N6-methyladenosine binding protein YTHDF2
Source: Mol Cancer. 2021 Mar 3;20:45. doi: 10.1186/s12943-021-01340-8 (PMC7927415; doi:10.1186/s12943-021-01340-8)
Supplement: Supplementary file 14 — Additional file 14: Table S1. shRNA sequences and primer sequences for RT-qPCR. [file 12943_2021_1340_MOESM14_ESM.docx]

Supplementary table 5: shRNA sequences and qRT‐PCR primer sequences

|  | sequences |
| --- | --- |
| FBXW7 shRNA1-F | CCGGACTTCTACTGTACGGTGTATGTTCAAGAGACATACACCGTACAGTAGAAGTTTTTG |
| FBXW7 shRNA1-R | AATTCAAAAAACTTCTACTGTACGGTGTATGTCTCTTGAACATACACCGTACAGTAGAAG |
| FBXW7 shRNA2-F | CCGGCCCATGGAAGAGTGCGTATATTTCAAGAGAATATACGCACTCTTCCATGGGTTTTTG |
| FBXW7 shRNA2-R | AATTCAAAAACCCATGGAAGAGTGCGTATATTCTCTTGAAATATACGCACTCTTCCATGGG |
| YTHDF2 shRNA1-F | CGGGGACGTTCCCAATAGCCAACTCTCGAGAGTTGGCTATTGGGAACGTCCTTTTTG |
| YTHDF2 shRNA1-R | AATTCAAAAAGGACGTTCCCAATAGCCAACTCTCGAGAGTTGGCTATTGGGAACGTCC |
| YTHDF2 shRNA2-F | CCGGAAGGACGTTCCCAATAGCCAACTCGAGTTGGCTATTGGGAACGTCCTTTTTTTG |
| YTHDF2 shRNA2-R | AATTCAAAAAAAGGACGTTCCCAATAGCCAACTCGAGTTGGCTATTGGGAACGTCCTT |
| FBXW7-F | TGG GAT ATC AAA ACA GGA CAG TGT |
| FBXW7-R | TAA ACA GGT CAC AGC ACT CTG ATG |
| YTHDF2-F | AGCCCCACTTCCTACCAGATG |
| YTHDF2-R | TGAGAACTGTTATTTCCCCATGC |
| BMF-F | GAGCCATCTCAGTGTGTGGAG |
| BMF-R | GCCAGCATTGCCATAAAAGAGTC |
| KCNK3-F | CTACGAGCACTGGACCTTCTT |
| KCNK3-R | CGTAAGGATGTAGACGAAGCTGA |
| MYBL2-F | CCGGAGCAGAGGGATAGCA |
| MYBL2-R | CAGTGCGGTTAGGGAAGTGG |
| LEMD1-F | ATTGCAGAACCAACTTGAGAAGC |
| LEMD1-R | CGCGCAGTAGTCTCTCTCTT |
| SIRPD-F | TCCATGTGCAACAAACGGAGA |
| SIRPD-R | GGTATTGGGTACGCTGCAACT |
| LY6K-F | ACGGACGAGGGTGACAATAGA |
| LY6K-R | CCGCTATAACGCAGTATGGC |
| SEMA3C-F | TTTGCGTGTTGGTTGGAGTAT |
| SEMA3C-R | TCCTGTAGTCTAAAGGATGGTGG |
| C1QTNF1-F | GTGCCCCAGATCAACATCACT |
| C1QTNF1-R | CCGTGTCGAAGATCACCGTC |
| ZDHHC14-F | ACAAGTTCTTCTGTAACGGGAGG |
| ZDHHC14-R | GAGTCCGCTAGTGACCAGGA |
| MOCS3-F | GAGGAGGTACTCGCCTTACAA |
| MOCS3-R | TCAGCCAAAAGAGCCGACG |
