# Supplementary material for: FBW7 suppresses ovarian cancer development by targeting the N6-methyladenosine binding protein YTHDF2
Source: Mol Cancer. 2021 Mar 3;20:45. doi: 10.1186/s12943-021-01340-8 (PMC7927415; doi:10.1186/s12943-021-01340-8)
Supplement: Supplementary file 15 — Additional file 15: Table S2. The relationship between FBW7 expression and the clinicopathological features of EOC. [file 12943_2021_1340_MOESM15_ESM.docx]

Supplementary table 1: Relationship between FBW7 expression and clinicopathologic factors of patients with epithelial ovarian cancer

| **Parameter** | **No. of patients** | **FBW7 expression** | | **P value** |
| --- | --- | --- | --- | --- |
|  |  | **low** | **high** |  |
| **Age** |  |  |  | 0.322 |
| ≤55 | 70 | 40 | 30 |  |
| >55 | 50 | 24 | 26 |  |
| **Tumor stage** |  |  |  | 0.922 |
| Ⅰ | 2 | 1 | 1 |  |
| Ⅱ | 10 | 5 | 5 |  |
| Ⅲ | 83 | 46 | 37 |  |
| IV | 25 | 12 | 13 |  |
| **Lymph node status** |  |  |  | 0.705 |
| Negative | 75 | 41 | 34 |  |
| Positive | 45 | 23 | 22 |  |
| **Ascites** |  |  |  | 0.806 |
| Negative | 33 | 17 | 16 |  |
| Positive | 87 | 47 | 40 |  |
| **Menopause** |  |  |  | 0.926 |
| YES | 63 | 37 | 26 |  |
| No | 57 | 33 | 24 |  |
