# Supplementary material for: FBW7 suppresses ovarian cancer development by targeting the N6-methyladenosine binding protein YTHDF2
Source: Mol Cancer. 2021 Mar 3;20:45. doi: 10.1186/s12943-021-01340-8 (PMC7927415; doi:10.1186/s12943-021-01340-8)
Supplement: Supplementary file 17 — Additional file 17: Table S4. The relationship between YTHDF2 expression and the clinicopathological features of EOC. [file 12943_2021_1340_MOESM17_ESM.docx]

Supplementary table 3: Relationship between YTHDF2 expression and clinicopathologic factors of patients with epithelial ovarian cancer

| **Parameter** | **No. of patients** | **YTHDF2 expression** | | **P value** |
| --- | --- | --- | --- | --- |
|  |  | **Low** | **High** |  |
| **Age** |  |  |  | 0.172 |
| ≤55 | 68 | 42 | 26 |  |
| >55 | 47 | 23 | 24 |  |
| **Tumor stage** |  |  |  | 0.262 |
| Ⅰ | 2 | 0 | 2 |  |
| Ⅱ | 9 | 6 | 3 |  |
| Ⅲ | 79 | 47 | 32 |  |
| IV | 25 | 12 | 13 |  |
| **Lymph node status status** |  |  |  | 0.410 |
| Negative | 71 | 38 | 33 |  |
| Positive | 44 | 27 | 17 |  |
| **Ascites** |  |  |  | 0.293 |
| Negative | 31 | 20 | 11 |  |
| Positive | 84 | 45 | 39 |  |
