# Supplementary material for: FBW7 suppresses ovarian cancer development by targeting the N6-methyladenosine binding protein YTHDF2
Source: Mol Cancer. 2021 Mar 3;20:45. doi: 10.1186/s12943-021-01340-8 (PMC7927415; doi:10.1186/s12943-021-01340-8)
Supplement: Supplementary file 18 — Additional file 18: Table S5. Correlations between the expression of FBW7/YTHDF2 and clinicopathological characteristics. [file 12943_2021_1340_MOESM18_ESM.docx]

Table 7 Correlations between the expression of FBW7/YTHDF2 and clinicopathological characteristics

| Variables | FBW7/YTHDF2 expression | | | | |  |
| --- | --- | --- | --- | --- | --- | --- |
|  | FBW7Low /YTHDF2 Low | | FBW7High /YTHDF2Low | FBW7Low /YTHDF2High | FBW7High /YTHDF2High | p-value |
| **Age** |  |  | |  |  |  |
| ≤55 | 24 | 18 | | 16 | 10 | 0.441 |
| >55 | 12 | 11 | | 12 | 12 |  |
| **Tumor stage** |  |  | |  |  |  |
| Ⅰ+Ⅱ | 4 | 2 | | 2 | 3 | 0.814 |
| Ⅲ+ IV | 32 | 27 | | 26 | 19 |  |
| **Lymphnode status** |  |  | |  |  |  |
| Negative | 21 | 17 | | 20 | 13 | 0.688 |
| Positive | 15 | 12 | | 8 | 9 |  |
| **Ascites** |  |  | |  |  |  |
| Negative | 11 | 9 | | 6 | 5 | 0.773 |
| Positive | 25 | 20 | | 22 | 17 |  |
| **Menopause** |  |  | |  |  |  |
| YES | 14 | 16 | | 12 | 10 | 0.614 |
| NO | 22 | 13 | | 16 | 12 |  |
