# Supplementary material for: FBW7 suppresses ovarian cancer development by targeting the N6-methyladenosine binding protein YTHDF2
Source: Mol Cancer. 2021 Mar 3;20:45. doi: 10.1186/s12943-021-01340-8 (PMC7927415; doi:10.1186/s12943-021-01340-8)
Supplement: Supplementary file 19 — Additional file 19: Table S6. Multivariate analysis of variables associated with PFS and OS. [file 12943_2021_1340_MOESM19_ESM.docx]

Table 6 Multivariate Cox regresion analysis with PFS and OS

| Factors |  | OS |  |  | PFS |  |
| --- | --- | --- | --- | --- | --- | --- |
|  | HR | 95%CI | p-value | HR | 95%CI | p-value |
| **Age** | 2.206e+08 | 0.1123-Inf | 0.9985 | 1.000e+00 | 0.3895-2.568 | 0.9999 |
| **Tumor stage** | 2.170e+08 | 0.2333-Inf | 0.9985 | 7.175e+00 | 0.9758-52.757 | 0.0529 |
| **Lymphnode status** | 9.353e-01 | 0.3053-2.865 | 0.9067 | 6.766e-01 | 0.3666-1.249 | 0.2114 |
| **Ascites** | 4.279e-01 | 0.1491-1.228 | 0.1146 | 9.989e-01 | 0.5081-1.964 | 0.9975 |
| **Menopause** | 4.515e-09 | 0.1432-Inf | 0.9985 | 1.046e+00 | 0.4085-2.679 | 0.9250 |
| **FBW7 expression** | 7.093e-01 | 0.2532-1.987 | 0.5133 | 6.958e-01 | 0.3797-1.275 | 0.2404 |
| **YTHDF2 expression** | 3.415e+00 | 1.0731-10.868 | 0.0376 | 9.690e-01 | 0.5383 -1.744 | 0.9163 |
